# Pathogen host jump risk is not predicted by spillover rate, but rather by novelty

**DOI:** 10.1371/journal.pbio.3003640

**Published:** 2026-03-19

**Authors:** Brandon J. Simony, David A. Kennedy

**Affiliations:** Department of Biology and the Huck Institutes of the Life Sciences, The Pennsylvania State University, University Park, Pennsylvania, United States of America; ETH Zurich, SWITZERLAND

## Abstract

Host jumps—defined as the process by which a pathogen establishes sustained transmission in novel hosts—are threats to human and animal welfare, but anticipating which pathogen will be the next to successfully host jump remains elusive. A spillover event must precede a host jump, and so spillover rate is thought to be related to risk. However, nonendemic pathogens that spill over frequently have demonstrated a poor ability to host jump from any given spillover. So which is riskier, pathogens that spill over rarely or commonly? Applying a Bayesian framework to a general model of host jump risk, we show that 1) the riskiest pathogens can be those that spill over at low, intermediate, or high rates, and 2) as the rate of spillover gets large, the information gained from past spillovers is exactly counterbalanced by the increased number of future spillovers. Taken together, this means that spillover rate has little to no value in explaining host jump risk. Rather, we show that novel pathogens (i.e., pathogens with a relatively short history of spilling over in their current form) are substantially more likely to result in host jumps than pathogens that have had long-associated opportunities for spillover into the novel host. Notably, a pathogen might be thought of as novel if spillover only recently became possible, or if it recently underwent substantial evolutionary change. We therefore propose that the length of historical association, but not spillover rate, will be an important predictor of host jump risk.

## Abstract 2

Host jumps pose major risks to health, but predicting them remains challenging due to complex spillover dynamics. This study shows that pathogen novelty, rather than spillover rate, is a stronger predictor of host jump risk, emphasizing the importance of monitoring emerging pathogens with limited spillover histories.

## Introduction

Zoonotic pathogens—defined as nonhuman animal pathogens that can infect humans—are a growing concern to global public health, and it is expected that they will be responsible for the next pandemic [1,2]. This is because most human pathogens are thought to have zoonotic origins [3–5]. Zoonotic pathogens can infect a human through spillover—defined as a pathogen transmission event from a native host species into a novel host species. Notably, spillover is not unique to humans or zoonotic pathogens, but rather can occur between any nonnative pathogen and any novel host. When the pathogen, following spillover, transmits well enough to establish itself indefinitely in the novel host population, we refer to this event as a host jump. A large body of research has focused on identifying characteristics that predispose pathogens to successfully host jump [5–15]. Spillover is a prerequisite to a host jump, and so efforts to reduce host jump risk have largely focused on pathogens that spill over frequently [16–20]. However, it is unclear how a pathogen’s inherent rate of spillover relates to the likelihood of a successful host jump. Put another way, are pathogens that frequently spill over more likely to host jump than those that spill over rarely?

At present, the answer to this question is unknown. The risk of a host jump undoubtedly depends on many system-specific details, including characteristics of the native and novel hosts, the biology of the nonnative pathogens, and numerous ecological and evolutionary factors. However, by considering two pathogens that differ only in their inherent rate of spillover, we can evaluate how spillover rate itself impacts host jump risk. On one hand, a pathogen that frequently spills over has many opportunities to complete a host jump, so all else equal, a pathogen that spills over more frequently may be conceivably more likely to successfully host jump. On the other hand, a pathogen that has not yet jumped hosts despite having spilled over many times has already demonstrated a record of failure [18,21], and so pathogens that have spilled over at high rates in the past may have a low likelihood of completing a host jump. Whether a pathogen with a high spillover rate is more likely to complete a host jump than a pathogen with a low spillover rate depends on how these two competing effects combine together.

To further illustrate the interplay between spillover rate and host jump likelihood, consider several examples. Rabies lyssavirus, the causative agent of rabies, is estimated to cause tens of thousands of human rabies cases per year due to zoonotic spillover from dogs and other animal reservoirs [22,23]. Rabies presents a significant public health burden, so preventive measures in place to mitigate spillover are undoubtedly important [24]. However, even without these preventive measures and despite the high rate of spillover, few would argue that rabies is a pandemic threat, given the limited opportunities for human-to-human transmission, which would presumably require a human biting another human [25]. A similar argument could be made for *Borellia burgdorferi*, the causative agent of Lyme disease, where sustained human transmission would be limited by the frequency and duration of ticks feeding on humans [26]. In contrast, human immunodeficiency virus (HIV) has completed host jumps into humans on at least two separate occasions [27], and these host jumps occurred after a presumably smaller number of spillover events than the aforementioned pathogens. Despite being a hand-picked list, these examples do illustrate the potential for a disconnect between spillover rate and host jump risk.

Here, we develop a conceptual model that provides a quantitative framework for evaluating the relationship between spillover rate and host jump risk. We derive an analytical solution to this model that depends on the rate of past and future spillover events, the duration of time over which spillover of that pathogen was possible in the past (past spillover window), the time in the future over which host jump risk is being assessed (future spillover window), and our prior uncertainty in the probability that a single spillover of that pathogen will result in a host jump. Using this analytical solution, we show that a pathogen’s host jump risk is highly insensitive to the pathogen’s inherent rate of spillover. Rather, the key factor that dictates host jump risk is the duration of the past spillover window. In other words, the greatest risk of a successful host jump is posed by pathogens that only recently gained opportunities to spill over into the novel host. In practice, this might mean that there was historical separation (geographic or otherwise) between the native and novel host, or that the pathogen has evolved in such a way that the outcomes of past spillovers are no longer informative about the outcomes of future spillovers.

## Model overview

**Fig 1:**
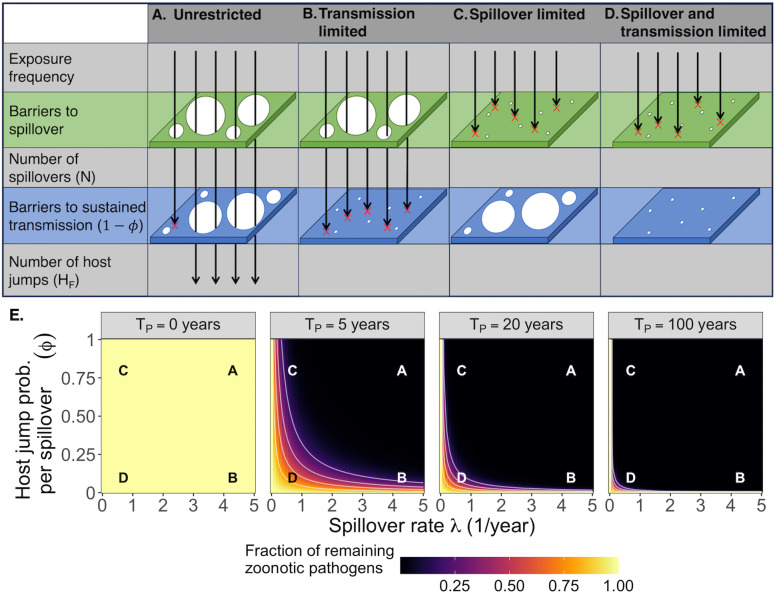
Conceptual framework for relating spillover rate and the past spillover window to host jump risk. In order to successfully host jump, a pathogen must overcome barriers to spillover and barriers to sustained transmission in the novel host [20,21]. Pathogens may or may not be limited at either step in this process, leading conceptually to four classes of nonnative pathogens (A–D). In practice, and in our model, spillover limitation and transmission limitation are continuous traits meaning that there is no discrete separation between the “types” of pathogens shown in A–D but we discuss pathogens in this framework because it is useful for illustration. In panel E, we show how the composition of the pathogen pool changes as the past spillover window $T_{P}\;$ increases (i.e., the amount of time a pathogen has had opportunities to spill over into the novel host). For illustrative purposes, we assume that all pathogens are equally frequent initially and that pathogens are removed when they complete a host jump. Since host jumps are more likely be completed in some parameter spaces than others, the pool of remaining pathogens (i.e., pathogens that have not yet completed a host jump) will change over time as a function of how spillover limited and transmission limited a pathogen is. As $T_{P}\;$ increases, the proportion of unrestricted pathogens (A) decreases quickly, meaning that as $T_{P}\;$ gets larger, pathogens are increasingly likely to be transmission limited (B), spillover limited (C) or both (D). Notably, the value of $T_{P}\;$ depends on both the host and pathogen and thus need not be the same for all nonnative pathogens. The code needed to generate panel E of this figure can be found in https://doi.org/10.5281/zenodo.14154724.

It is worth acknowledging that despite all the complexities of biology, a successful host jump can be exactly distilled into a simple two-part process. First, a pathogen must spill over into a novel host thereby infecting it, and second, the pathogen must spread sufficiently well as to establish sustained transmission in the novel host population. To complete a host jump, pathogens must overcome barriers in both of these processes [20]. Conceptually, pathogens can therefore be broadly divided into four categories based on their ability to overcome each of these barriers: “unrestricted”, “transmission limited”, “spillover limited”, and “spillover and transmission limited” (Fig 1A–1D). We define “unrestricted pathogens” as those that frequently spill over and are also likely to sustain transmission in the novel host population. “Transmission limited” pathogens are those that frequently spill over but are unlikely to sustain transmission in the novel host. “Spillover limited” pathogens are those that rarely spill over but are likely to sustain transmission in the novel host if they do. “Spillover and transmission limited” pathogens are those that rarely spill over and are also unlikely to sustain transmission in the novel host population.

The proportion of pathogens that fall within each of these categories is likely to change over long periods of time, in part because once a host jump occurs, the pathogen that jumped hosts changes from being a nonnative pathogen to being an endemic pathogen of foreign origin. Fig 1E illustrates how the composition of nonnative pathogens changes over time, or more accurately, how it changes as a function of how long there have been opportunities for each pathogen to spill over into the novel host. We refer to this duration as the past spillover window $T_{P}\;$, where large values mean the pathogen has had a long historical association with the novel host. Note that the change in the prevalence of a particular pathogen depends on both how spillover-limited and transmission-limited it is, respectively, the x and y axes. For clarity and without loss of generality, we assume that $T_{P}\;$ is measured in units of years and the spillover rate λ is measured in units of spillover events per year. Note that as $T_{P}\;$ increases, unrestricted nonnative pathogens quickly become rare because any that initially existed would quickly complete host jumps. Thus at any given time, most nonnative pathogens will be spillover limited, transmission limited, or both. Note, however, that $T_{P}\;$ will differ for different pathogens depending on how long each has had opportunities for spillover in its current form.

To develop a model of host jump risk, it is first useful to realize that there are only two possible outcomes that can occur following a spillover event; either the pathogen dies out in the novel host population or it establishes itself. Although the precise disease dynamics that arise following a spillover event will depend on a complicated demographic process, the outcome can be modeled as a coin flip, where the probability of “heads” (defined as ϕ , or equivalently the chance that a single spillover will result in a host jump) is the probability of nonextinction following a spillover event. Thus, assuming independence between spillover events, the probability that none of N spillover events result in a host jump (i.e., P(H=0) is simply $(1-\phi {)}^{N}\;$. As an aside, each spillover event presumably has a unique host jump probability $(\phi_{j}$ that will depend on numerous host, pathogen, and environmental traits. However, our equation is unchanged even in the presence of this variation, provided ϕ  is set to the average of $\phi_{j}\;$, and the average of $\phi_{j}\;$ does not change over time (Supporting information S1 Text).

Moving from counts of spillover events to rates of spillover events, if we assume the risk of spillover is constant, we can analogously define the probability that no host jump occurs within a time window of length T using a Poisson process with parameter $\begin{array}{c} \lambda T\; \end{array}$. Here $\begin{array}{c} \lambda \end{array}$ is the pathogen’s rate of spillover and T is the time interval over which the probability of a host jump is being considered. When the outcome of each spillover event is independent, the probability that no host jump occurs within a time window of length T is


$$P(H=0|\lambda ,T,\phi )=e^{-\phi \lambda T}.\;$$ (1)

One of the key challenges with using this equation, however is that ϕ  (the probability of persistence for a single spillover event) is typically unknown. Here, we propose that, by employing a Bayesian framework, the past can be used to inform the value of ϕ . To illustrate our logic, consider a pathogen that has spilled over millions of times in the past, but has not yet completed a host jump. Intuitively, its value of ϕ  must be very small, since it likely would have already jumped hosts otherwise.

This logic can be formalized by treating ϕ  as a distribution rather than a fixed constant, where π(ϕ is the distribution that describes the plausible values of ϕ  (in Bayesian statistics, this is referred to as a prior). One advantage of this approach is that it explicitly allows our uncertainty about the value of ϕ  to be updated in a statistically rigorous way as new data become available. Following the definition of Bayes’ theorem, our posterior distribution of ϕ  is simply


$$\pi (\phi |\lambda ,T_{P},H_{P}=0)=Z\cdot \pi (\phi )\cdot e^{-\phi \lambda T_{P}}\;$$ (2)

where *Z* is a posterior normalizing constant, π(ϕ is again our prior, and the exponential term is the likelihood of having no host jumps occur in the time window $T_{P}\;$. The subscript *P* denotes that parameters are referring to the past. Note two key assumptions required for the above equation to be exactly correct. First, we assume that ϕ  is constant over time. Second, we assume we are modeling a world in which there is either only a single “pathogen” (however broadly or narrowly that term might be defined) or the value of ϕ  for each “pathogen” is independent of the others. We readily acknowledge that these assumptions will be universally violated when our model is applied to real systems, but this conceptually simplified model yields fundamental insights that are robust to these assumptions.

We can then use Eq. 2 to quantify future host jump risk for a particular theoretical pathogen in a particular time window of size $T_{F}\;$. This probability can be expressed as:


$$P(H_{F}>0|•,H_{P}=0)=1-Z\int_{0}^{1}\pi (\phi )\cdot e^{-\phi \lambda (T_{P}+cT_{F})}d\phi \;$$ (3)

Above, **•** represents the model parameters. Host jump risk thus depends on the rates of past and future spillover λ and cλ  respectively, the size of the past and future spillover windows $T_{P}\;$ and $T_{F}\;$ respectively, and our prior belief about the likelihood that a given spillover event will result in a host jump π(ϕ. Note that although in the present analyses we only consider cases where no past spillover event resulted in a host jump $(\text{i.e., }H_{P}=0$, this equation can be readily extended—although perhaps less usefully—to cases where past spillovers have resulted in successful host jumps (Supporting information S2 Text).

To finish parameterizing our model, it is necessary to define π(ϕ, which reflects our initial uncertainty regarding the probability that a single pathogen spillover event will result in a host jump. Note that the shape of this distribution would presumably depend on the definition of “pathogen” being used, which could in theory be anything from a single clonal genotype to the set of all microbes worldwide. Regardless of the definition, however, specifying an appropriate shape for this distribution is not straightforward. We therefore consider three possible shapes for our prior distribution. Notably, one could argue for a range of possible shapes. The following three were chosen to illustrate the role that the prior’s shape can have on host jump risk.

**Fig 2:**
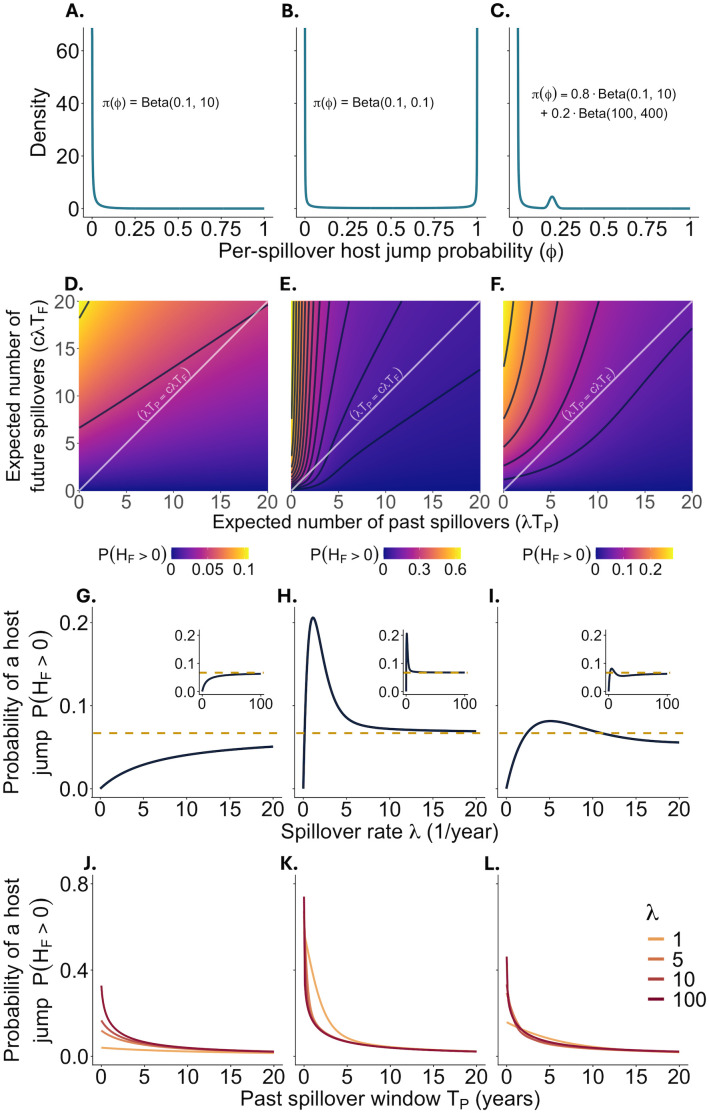
Host jump risk is strongly dependent on the past spillover window $\begin{array}{c} T_{P} \end{array}$ but not the spillover rate $\begin{array}{c} \lambda \end{array}$. The columns of panels from left to right denote Scenarios 1-3 respectively. Panels A-C show the prior distributions on ϕ . Panels D-F show the model-calculated probability of a successful host jump as determined from Eq. 4. Axes show the effects of changing the two key parameter combinations, which relate to the expected number of past spillover events and the expected number of future spillover events. Black contour lines demarcate 5% intervals in overall host jump risk. Note that the color range differs for each panel. To explore the impact of a pathogen’s inherent spillover rate λ on host jump risk, we can explore how host jump risk changes along a straight line where the slope of the line is $c\frac{T_{F}}{T_{P}}\;$. The white line depicts the case where *T*_F_ = *T*_P_ = 1 and c=1 . Panels G-I depict probabilities along this line, and they reveal that depending on the prior π(ϕ, host jump risk may be greatest at high, low, or intermediate rates of spillover. Nevertheless, in all scenarios, host jump risk converges as spillover rate increases (Eq. 5, dashed gold line, inset figures) demonstrating that as a pathogen’s inherent rate of spillover increases, the rate gives progressively less information about host jump risk. In panels J-L, we plot the probability of a future host jump as a function of the past spillover window *T*_*P*_ (shown for *T*_*F*_ = 5). This is equivalent to moving horizontally in panels D-F. In each scenario, the probability of a host jump declines monotonically and dramatically as *T*_*P*_ increases from zero, demonstrating that the greatest risk of a host jump is posed by pathogens that are new, as assessed from the perspective of the novel host. Conceptually, we could think of a pathogen as “new” if the novel host was never previously in a setting where spillover was possible, or if the pathogen had evolved in such a way that it was now effectively a different pathogen than in the past. Furthermore, these panels (J-L) show that even with drastically different rates of spillover, host jump risk is much more strongly impacted by changes in the size of the past spillover window *T*_*P*_ than changes in the rate of spillover λ, adding support to our claim that spillover rate is not a useful predictor of host jump risk. In all panels when not specified, c=1 , $T_{P}=T_{F}=1\;$ year. The code needed to generate this figure can be found in https://doi.org/10.5281/zenodo.14154724.

1. A nonnative pathogen will be unlikely to persist in the novel host, meaning the distribution of ϕ  is extremely right-skewed (Fig 2A).
2. A nonnative pathogen may fall into either of two distinct sub-classes, where it has either a low or high likelihood of persisting in the novel host, meaning that the distribution of ϕ  is “U-shaped” (Fig 2B).
3. A nonnative pathogen may fall into either of two distinct sub-classes, where it has either a low or intermediate likelihood of persisting in the novel host, meaning the distribution of ϕ  is multi-modal with one peak at 0 and another peak at an intermediate value (Fig 2C).

Scenario 1 and 2 above can be modeled using a beta distribution and Scenario 3 can be modeled using a mixture of two beta distributions. The results of our analyses are general, but for illustration purposes, we will specify parameter values for each of our prior distributions such that in Scenario 1: π(ϕ)∼Beta(a=0.1,b=10, in Scenario 2: π(ϕ)∼Beta(a=0.1,b=0.1, and in Scenario 3: $\pi (\phi )∼\omega_{1}\text{Beta}(a=0.1,\;b=10)+\omega_{2}\text{Beta}(a=100,\;b=400);\;\omega_{1}=0.8,\;\omega_{2}=0.2\;$. In Supporting information S2 Text, we show that when π(ϕ follows a beta distribution, there is an analytical solution to Eq. 3 such that:


$$\begin{array}{c} P(H_{F}>0|•,H_{P}=0)=1-\frac{ℳ(a,\;a+b,-\lambda (T_{P}+cT_{F}))}{ℳ(a,\;a+b,-\lambda T_{P})} \end{array}\;$$ (4)

where **∙** again represents the set of model parameters and ℳ(x,y,z) is the confluent hypergeometric function of the first kind [28]. In Supporting information S2 Text, we derive a similar analytical solution for beta-mixture distributions like Scenario 3, and in Supporting information S3 Text, we show that all of our main results hold for a model parameterized based on counts rather than rates of spillover.

While the functional form of Eq. 4 may not immediately yield intuitive insights, it can be readily evaluated to determine how host jump risk is influenced by the various model parameters. In addition, examination of Eq. 4 shows that while the risk of a host jump depends on a total of six parameters, it depends on only four parameter combinations: the hyperparameter *a* and its sum with the hyperparameter *b* (which determine the shape of π(ϕ), the expected number of past spillover events (which is the spillover rate λ times the past spillover window $T_{P}\;$), and the expected number of future spillover events (which is the future spillover rate cλ  times the future spillover window over which host jump risk is being assessed $T_{F}\;$). In what follows, we use Eq. 4 and its beta-mixture analog from Supporting information S2 Text to show that host jump risk is largely insensitive to spillover rate λ but is highly sensitive to the length of time that spillover was possible in the past $T_{P}\;$, thus demonstrating that the length of historical association, rather than spillover rate, is the key driver of host jump risk. All main results were also confirmed using simulation (Supporting information S4 Text).

## Results

Using Eq. 4, we evaluate the probability of a successful host jump in scenarios 1–3 (Fig 2 panels D-F respectively) as a function of the key parameter combinations $\lambda T_{P}\;$ (expected number of past spillovers) and $c\lambda T_{F}\;$ (expected number of future spillovers). In all three scenarios, when only varying the future values, the probability of a future host jump increases as the expected number of future spillover events $c\lambda T_{F}\;$ increases (i.e., host jump probability increases when moving from bottom to top in Fig 2D–2F). Intuitively, this should be true, as more spillover events create more opportunities for a host jump to occur. In contrast, when only varying the past values, the probability of a future host jump decreases as the expected number of past spillover events increases (i.e., host jump probability decreases when moving from left to right in Fig 2D–2F). This can be explained by the increased observation of failed host jumps driving the posterior $\pi (\phi |\lambda ,T_{P},H_{P}=0$ closer to zero, making each future spillover event less likely to result in a host jump. In what follows, we delve deeper into these results to separate the impact of spillover rate λ from the impact of time, specifically $T_{P}\;$.

With regard to spillover rates, the past and future are often correlated. Pathogens with high past spillover rates are likely to have high future spillover rates, and pathogens with low past spillover rates are likely to have low future spillover rates. We have implicitly assumed this linear relationship by defining the future spillover rate as cλ , where *c* is an arbitrary constant that accounts for differences between the past and future due to changes outside the pathogen’s control (we relax the assumption of linearity in Supporting information S5 Text). Using this formulation, we can then assess the relationship between a pathogen’s inherent spillover rate and its risk of a future host jump. Holding $T_{P}\;$ and $T_{F}\;$ constant, this means that as a pathogen’s inherent rate of spillover changes, it moves along Fig 2 panels D-F on a straight line with slope $c\frac{T_{F}}{T_{P}}\;$ (or equivalently *c* when $T_{F}=T_{P}\;$). By comparing host jump risk along this line, we can therefore assess the role of a pathogen’s inherent rate of spillover on its host jump risk.

For illustrative purposes, we show the case where the past and future spillover rates are equal (c=1, but note that all of our results are qualitatively similar for other linear correlations. Fig 2G–2I shows how the probability of a host jump varies for pathogens that are in all ways identical except for differences in their inherent rates of spillover (in other words, they have the same values for *a*, *b*, *c*, $T_{P}\;$, and $T_{F}\;$). We find that the spillover rate where host jump risk is greatest depends on the prior π(ϕ. In Scenario 1 (Fig 2G), increasing the spillover rate leads to a monotonic increase in host jump probability. In Scenario 2 (Fig 2H), host jump risk peaks at a low spillover rate and then decreases monotonically. In Scenario 3 (Fig 2I), host jump risk peaks at an intermediate spillover rate, declines at slightly higher rates, and then increases again when spillover rates become very high. These results therefore illustrate that, when comparing across pathogens, even with all else equal, pathogens with high rates of spillover may be either more or less likely to cause a host jump than pathogens with low rates of spillover, and the conclusion depends on both our prior distribution on ϕ  and the precise rates of spillover being compared.

Next, we ask how the probability of a host jump changes as the rate of spillover becomes large. To do so, we evaluate the limit of Eq. 4 as λ approaches infinity. In Supporting information S5 Text, we show that the probability of a host jump converges to


$$P(H_{F}>0|•,H_{P}=0)=1-\frac{1}{(c\frac{T_{F}}{T_{P}}+1{)}^{a}}.\;$$ (5)

Notably, Eq. 5 is strictly between zero and one when *a*, *c*, $T_{P}\;$ and $T_{F}\;$ are positive, meaning that host jump risk will converge to an intermediate value as the inherent rate of spillover goes to infinity. In Supporting information S5 Text, we derive the exact same solution for priors that use mixtures of beta distributions, where the value of *a* above is equal to the component of the beta mixture with the smallest *a*. Because the beta shape parameter *a* is the same for the three scenarios used here, all converge to the same value (the dashed gold lines in Fig 2G–2I). However, they can approach this value from below (i.e., higher rates of spillover lead to an increase in host jump risk) or above (i.e., higher rates of spillover lead to a decrease in host jump risk). This implies that our uncertainty regarding the shape of π(ϕ can fundamentally change our conclusion as to whether the greatest host jump risk comes from pathogens that spill over frequently or those that spill over rarely. In addition, Fig 2G–2I demonstrates that, except at very low rates of spillover, host jump risk changes very slowly relative to the change in spillover rate, meaning that large differences in spillover rate between two pathogens will typically have little influence on the overall probability that a host jump will occur.

We next address how the past spillover window $T_{P}\;$ relates to the probability of a successful host jump. Note that in contrast to spillover rate, there is no reason to expect $T_{P}\;$ to correlate with $T_{F}\;$ (the time window over which the risk of a future host jump is being assessed). Therefore, in analyzing these results, we assume $T_{F}\;$ is invariant with regard to $T_{P}\;$. Functionally, this is equivalent to moving left or right on Fig 2D–2F. In Fig 2J–2L, we plot the probability of a future host jump as a function of the past spillover window $T_{P}\;$ for each of our three priors. This figure shows that host jump risk is strongly affected by $T_{P}\;$, declining rapidly and monotonically as $T_{P}\;$ increases. This claim is reinforced by Eq. 5, which shows that the probability of a host jump goes to zero as $T_{P}\;$ gets large. The figure also reiterates our earlier claim that the rate of spillover only has a minimal effect on host jump risk, and it demonstrates that any variation in host jump risk attributable to differences in spillover rates becomes less important as $T_{P}\;$ increases. Thus, the pathogens most likely to host jump are not those that spill over frequently, but rather those that have not had a long prior association with the novel host. The one caveat here is that for pathogens where $T_{P}\;$ is exactly equal to 0 (i.e., pathogens that have never yet had any opportunity for spillover), higher rates of spillover are universally worse. All together, our results indicate that a pathogen’s inherent rate of spillover is not predictive of host jump risk, but rather, the length of historical association is.

## Discussion

Here we have derived a general framework to characterize the relationship between the spillover rate of a pathogen and the probability of that pathogen successfully emerging in (“jumping into”) a novel host. Using this framework, we show that conditional on a pathogen having not yet successfully completed a host jump by a given point in time, there is not a predictable relationship between spillover rate λ and future host jump risk. Rather, the direction of the relationship depends on the prior distribution of ϕ  (equivalently, our uncertainty in the probability that a single spillover event will result in a successful host jump). Moreover, we show that changes in spillover rate have progressively less impact on host jump risk as spillover rate increases, meaning that there is negligible difference in risk between pathogens with moderate or high rates of spillover (Fig 2), noting that the definition of “moderate” depends on the hyperparameters of the prior (see Supporting information S6 Text). Finally, we show that in contrast to spillover rate, the risk of a host jump quickly decreases as the past spillover window $T_{P}\;$ (i.e., the amount of time that pathogen has had opportunities to spill over) increases. In other words, pathogens that have only recently started associating with a novel host pose a greater risk for host jumps than ones that have had longer associations, while pathogens that spill over a lot are not inherently more or less risky than those that spill over rarely. We thus conclude that the spillover rate of a pathogen is not useful for predicting the future risk of a host jump but that the risk depends strongly on the size of the past spillover window.

Perhaps counterintuitively, our model illustrates that host jump risk may be maximized at high, low, or intermediate rates of spillover (Scenarios 1, 2, and 3 respectively). As revealed in Fig 2D–2F and Eq. 4, the host jump risk posed by a pathogen depends on the prior of ϕ . In other words, the answer we give to the question of which pathogen is riskiest depends on our initial belief about the probability that a given spillover event will result in a host jump (see Supporting information S6 Text for more discussion of how the shape parameters *a* and *b* affect the relationship between spillover rate and host jump risk). Consequently, if two independent decision makers disagreed on whether Scenario 1 (all pathogens are unlikely to jump hosts) or Scenario 3 (a subset of pathogens are risky) is more realistic, they would arrive at fundamentally different conclusions as to which pathogens are riskiest. We note, however, that these findings do not dispute the conventional wisdom that spillover reduction decreases host jump risk. This is because spillover reduction efforts reduce spillover in the future but do not affect the past (i.e., equivalent to moving from top to bottom in Fig 2D–2F.) It is additionally worth noting that spillover reduction provides public health benefits beyond simply reducing host jump risk [24,29].

A key point revealed by our model is that host jump risk is highly sensitive to the size of the past spillover window $T_{P}\;$, which represents the length of historical association between the pathogen and novel host (Fig 2J–2L). In particular, pathogens that only recently gained opportunities to spill over are riskier than ones with long-established histories. Fig 2J–2L shows that this conclusion is robust to the choice of prior, demonstrated by the fact that host jump risk monotonically decreases as the past spillover window $T_{P}\;$ increases in each scenario. This can be explained using the same underlying logic we used to explain why spillover rate is not predictive of host jump risk; pathogens that have proven themselves to be poorly suited to complete a host jump in the past are unlikely to successfully host jump in the future. Notably, however, our model assumes that the pathogen population does not change over time, whereas in reality, pathogen populations typically evolve over time even in their reservoir hosts. If such evolution were to occur, the outcomes of past spillover events might be only partially informative about the risk posed by future spillover events [9,30]. Our model could, in theory, be re-imagined to fit this scenario by shortening the past spillover window $T_{P}\;$ to the period over which the pathogen population was “substantially similar” to its current form. However, to precisely capture the true host jump risk, as might be desired if our model were being applied to real-world zoonotic pathogens, a much more complicated version of Eq. 2 would be needed that accounts for the gradual loss of information attributable to pathogen evolution.

Previous studies addressing host jump risk have largely revolved around identifying underlying drivers of host jump risk based on correlative approaches [21,31–34]. Here, we have proposed a model framework utilizing tools from Bayesian statistics to infer how host jump risk changes as a function of the pathogen’s rate of spillover and its past spillover window, and we thus refer to our approach as a mechanistic model. While mechanistic models have been used extensively for predicting the timing and location of spillover events [17,35–37], we show that a mechanistic approach can also provide insight into host jump risk. Notably, the current form of our model cannot yet be applied to real data, in large part because of difficulties in estimating the spillover rate λ and the prior π(ϕ. Incorporating more system-specific biological details into the prior or considering correlations between parameters (e.g., λ and ϕ ) could serve to further improve this approach in the future [10,21,31,34,38,39]. For instance, features such as phylogenetic or genomic data [14,40–42], host-pathogen characteristics [7,43–45], evolutionary theory [46–48], and the basic reproductive number $R_{0}\;$ [31,46] could all be integrated into characterizing our prior uncertainty of ϕ . However, even with these changes, the current form of our model would be inappropriate for real-world prediction because the posterior distribution in our model (Eq. 2) assumes that we are able to precisely define static pathogen populations that act independently from one another. In reality, pathogens are related to each other, and so the spillover of one pathogen would presumably inform the risk posed by a different, but related pathogen, thus implying a need to modify Eq. 2. However, these limitations do not undermine the insights generated by our model with regard to the role of spillover on pandemic prediction.

Our model results depend on three basic assumptions, namely: 1) the outcome of all spillover events are independent of one another, 2) the average probability that a spillover event results in a host jump (ϕ is constant over time, and 3) the rates of spillover in the past and future are linearly correlated. Each of these assumptions could be violated in certain situations. Our first assumption may be violated if spillover were so frequent that it led to high levels of immunity in the novel host population. This effect would disproportionately impact pathogens with high rates of spillover, presumably decreasing the likelihood of a host jump relative to our predictions. Our second assumption, that ϕ  is constant over time, could be violated due to changes in factors including host population density, connectivity, behavior, immunity, the locations of spillover events, or pathogen transmissibility due to host or pathogen evolution [49]. If ϕ  were to increase over time, the outcomes of past spillover events would provide less information about the risk posed by future spillover events than currently assumed in our model, and we therefore suspect that pathogens with high rates of spillover may pose a relatively greater threat than those with low rates of spillover, but this conclusion would likely depend on the precise way that ϕ  is changing over time. Our third assumption that past and future spillover rates are linearly correlated seems likely to hold in most cases. If this assumption were violated, however, the probability of a host jump would no longer converge to a value between zero and one (dashed gold line in Fig 2G–2I) as the rate of spillover goes to infinity, and would instead converge to 0 or 1, depending on whether the correlation was sub-linear or greater than linear (see Supporting information S5 Text).

Our model suggests that the relationship between spillover rate and host jump risk can be counter-intuitive: the pathogen that spills over most is not necessarily the greatest host jump threat, nor is it the case that exceptionally rare pathogens pose the greatest risk. Rather, the pathogens that pose the greatest risk of a host jump are those that are new from the perspective of the novel host, meaning that opportunities for spillover only recently became possible (noting that a “new” pathogen may also be an evolved version of an “old” pathogen). Efforts to mitigate future spillover will continue to be a powerful pandemic prevention tool [29], but our framework shows that the inherent spillover rate of a pathogen is a poor predictor of host jump risk.

## Supporting information

S1 Text Accounting for uniqueness of individual spillover events. (PDF)

S2 Text Poisson model derivation. (PDF)

S3 Text Count model. (PDF)

S4 Text Model validation using simulations and count parameterization. (PDF)

S5 Text Analytical solution to the limit (Poisson model). (PDF)

S6 Text Evaluating reasonable prior hyper-parameters. (PDF)
