# Supplementary material for: Pathogen host jump risk is not predicted by spillover rate, but rather by novelty
Source: PLoS Biol. 2026 Mar 19;24(3):e3003640. doi: 10.1371/journal.pbio.3003640 (PMC13001934; doi:10.1371/journal.pbio.3003640)
Supplement: S1 Text — (PDF) [file pbio.3003640.s001.pdf]

S1 Text. Accounting for uniqueness of individual spillover events

In the main text, we presented Eq. 1, which describes the probability that no host jumps occur in some length of time with a fixed rate of spillover λ. In that equation, we assumed that every spillover event had the same probability ϕ of resulting in a host jump. Here we show that relaxing the assumption that all spillover events have the same probability of resulting in a host jump has no impact on our model.

We define ϕ_j as the probability that some spillover event j results in a successful host jump. The value of a given ϕ_j depends on host-pathogen-environment interactions as well as temporal and individual level variation that might affect the likelihood of a host jump. For example, individuals may differ in their probability of becoming infected or in their transmissibility, so the particular individuals that become infected may change the probability that the given spillover event results in a host jump. Additionally, the location where spillover occurs may significantly influence host jump risk (e.g., rural vs. urban location). Not all values of ϕ_j are equally likely, so a given value of ϕ_j can be theoretically characterized as a random draw from some distribution Φ with density function $f_\Phi(\phi_j)$. Therefore, the probability that a single spillover event $(N = 1)$ results in a successful host jump ($H > 0$, or more specifically here $H = 1$) can be quantified as the product of ϕ_j and the probability density function for this particular value of ϕ_j, integrated over all possible values of ϕ.

$$P(H > 0|N = 1, \Phi) = \int_0^1 \phi_j \cdot f_\Phi(\phi_j) d\phi_j \tag{S1.1}$$

$$= E(\Phi) = \phi \tag{S1.2}$$

We see that this is the definition of the expected value (or mean) of a distribution, and we define ϕ as the mean of Φ. If we expand this to any number of spillover events, the probability that N spillover events fail to result in a host jump $(H = 0)$ where the vector of probabilities that the jth spillover event results in a host jump $(\vec{\phi_j})$ can be written as:

$$P(H = 0|N = 1, \vec{\phi_j}) = \prod_{j=1}^{N} (1 - P(H > 0|N = 1, \phi_j)) \tag{S1.3}$$

$$= \prod_{j=1}^{N} (1 - \phi_j) \tag{S1.4}$$

Due to the stochastic nature of spillover and the ensuing disease dynamics, it is effectively impossible to know the true ϕ_j for a given spillover event, so we instead multiply Eq. S1.4 by the probability that ϕ_j takes a particular value and integrate over all values of ϕ_j. Because we assume that each spillover event is independent, we can apply this to each instance of spillover individually such that

$$P(H = 0|N, \Phi) = \prod_{j=1}^{N} \int_0^1 (1 - \phi_j) \cdot f_\Phi(\phi_j) d\phi_j \tag{S1.5}$$

$$= \prod_{j=1}^{N} \int_0^1 f_\Phi(\phi_j) d\phi_j - \int_0^1 \phi_j \cdot f_\Phi(\phi_j) d\phi_j \tag{S1.6}$$

Using the definition of ϕ from Eq. S1.2 and the fact that $f_\Phi(\phi_j)$ is a proper density function and therefore integrates to 1 over its support, we get

$$P(H=0|N,\Phi) = \prod_{j=1}^{N} 1-\phi \tag{S1.7}$$

$$P(H=0|N,\Phi) = (1-\phi)^N \tag{S1.8}$$

We see that this equation exactly describes the binomial probability of observing no host jumps in some number of spillover events (N), if we assume that the mean of all possible values of ϕ_j does not change over time. Since this equation holds for all positive integer values of N, we can then use the law of total probability to expand this equation so that the number of spillover events in a given amount of time is stochastic. If we explicitly assume that the number of spillover events in some time interval (T) follows a Poisson process where λ is the pathogen's rate of spillover, we arrive at equation 1, and we show this derivation in Supporting information S2 Text. Therefore, we may equivalently express the probability of no host jumps ($H = 0$) for a pathogen with spillover rate λ in some time interval t when each spillover event has a unique probability of resulting in a host jump ϕ_j as a function of the mean of all possible values of ϕ_j, which we refer to as ϕ.
