# Supplementary material for: Pathogen host jump risk is not predicted by spillover rate, but rather by novelty
Source: PLoS Biol. 2026 Mar 19;24(3):e3003640. doi: 10.1371/journal.pbio.3003640 (PMC13001934; doi:10.1371/journal.pbio.3003640)
Supplement: S2 Text — (PDF) [file pbio.3003640.s002.pdf]

S2 Text. Poisson model derivation

In the main text, we presented a model for the probability of a successful host jump in some future spillover window (T_F) that depends on a pathogen's inherent rate of spillover (λ), the past spillover window (T_P), and the probability that an individual spillover event results in a successful host jump (ϕ). Here, we present a full generalized derivation of this model and provide an analytical solution for the case where the prior on ϕ follows a beta or mixture of beta distributions.

In what follows, we will derive a model for the probability of at least one successful host jump ($H_F > 0$) occurring in a fixed future spillover window (T_F) as a function of a pathogen's rate of spillover (λ), the past spillover window (T_P), the number of past host jumps (H_P), and our uncertainty in the probability that a spillover results in a successful host jump $\pi(\phi)$. We make the same assumptions as in the main text that the outcome of each spillover event is independent of all others, and that ϕ is not changing over time. Here, we assume that the number of spillover events in the past and future follow Poisson processes with rates λT_P and $c\lambda T_F$ respectively, so the number of spillovers in the past (N) and future (M) can be expressed as

$$N \sim Pois(\lambda T_P) \tag{S2.1}$$

$$M \sim Pois(c\lambda T_F) \tag{S2.2}$$

Equation 1 in the main text quantifies the probability that no host jumps occur (i.e., $H_P = 0$), conditional on λ, T_P, and ϕ. This can be generalized to reflect the probability that exactly H_P host jumps occur, and this can be derived using the law of total probability such that

$$P(H_P|\lambda, T_P, \phi) = \sum_{N=H_P}^{\infty} P(H_P|\phi, N) \cdot P(N|\lambda, T_P) \tag{S2.3}$$

$$P(H_P|\lambda, T_P, \phi) = \frac{1}{C_{norm}} \sum_{N=H_P}^{\infty} \binom{N}{H_P} \phi^{H_P} (1-\phi)^{N-H_P} \cdot \frac{(\lambda T_P)^N e^{-\lambda T_P}}{N!} \tag{S2.4}$$

$$C_{norm} = 1 - \sum_{k=0}^{H_P-1} \frac{(\lambda T_P)^k e^{-\lambda T_P}}{k!} \tag{S2.5}$$

where C_{norm} is a normalizing constant, which is necessary due to the constraint that $N \geq H_P$, and is only necessary when $H_P > 0$. We can continue to simplify the infinite sum in Eq. S2.5 such that

$$P(H_P|\lambda, T_P, \phi) = \frac{e^{-\lambda T_P} (\phi \lambda T_P)^{H_P}}{C_{norm} \cdot H_P!} \sum_{N=H_P}^{\infty} \frac{(\lambda T_P - \phi \lambda T_P)^{N-H_P}}{(N-H_P)!} \tag{S2.6}$$

$$= \frac{e^{-\lambda T_P} (\phi \lambda T_P)^{H_P}}{C_{norm} \cdot H_P!} \sum_{N-H_P=0}^{\infty} \frac{(\lambda T_P - \phi \lambda T_P)^{N-H_P}}{(N-H_P)!} \tag{S2.7}$$

$$= \frac{e^{-\lambda T_P} (\phi \lambda T_P)^{H_P}}{C_{norm} \cdot H_P!} \cdot e^{\lambda T_P - \phi \lambda T_P} \tag{S2.8}$$

Following these simplifying steps, we use the fact that the sum has the form $\sum_{n=0}^{\infty} \frac{x^n}{n!}$, which is the series definition of the exponential function. Using this property and simplifying this expression further, we get:

$$P(H_P|\lambda, T_P, \phi) = \frac{(\phi \lambda T_P)^{H_P}}{C_{norm} \cdot H_P!} \cdot e^{-\phi \lambda T_P} \tag{S2.9}$$

Equation S2.9 therefore gives a generalized form of Eq. 1 from the main text that allows for a non-zero number of host jumps in the past spillover window T_P, given some rate of spillover (λ) and the probability that a spillover results in a host jump (ϕ). We can then use this equation to derive our model when the number of past and future spillover events are Poisson distributed with a fixed rate. Using Eq. S2.9 in Bayes' theorem and simplifying, the posterior distribution can be expressed as:

$$\pi(\phi|\lambda, T_P, H_P) = \frac{\pi(\phi) \cdot P(H_P|\lambda, T_P, \phi)}{\int_0^1 \pi(\phi) \cdot P(H_P|\lambda, T_P, \phi) d\phi} \tag{S2.10}$$

$$\pi(\phi|\lambda, T_P, H_P) = \frac{\pi(\phi) \cdot \frac{(\phi\lambda T_P)^{H_P}}{C_{norm} \cdot H_P!} e^{-\phi\lambda T_P}}{\int_0^1 \pi(\phi) \cdot \frac{(\phi\lambda T_P)^{H_P}}{C_{norm} \cdot H_P!} e^{-\phi\lambda T_P} d\phi} \tag{S2.11}$$

$$\pi(\phi|\lambda, T_P, H_P) = Z \cdot \pi(\phi) \cdot e^{-\phi\lambda T_P} \phi^{H_P} \tag{S2.12}$$

In general, it is not possible to analytically evaluate the normalizing constant Z for any particular prior, so numerical integration is often required to determine this normalizing constant. However, when the prior follows a beta distribution, this constant Z can be expressed in terms of the confluent hypergeometric function [1] and the posterior can be written as

$$\pi(\phi|\lambda, T_P, H_P) = \frac{\pi(\phi) \cdot e^{-\phi\lambda T_P} \phi^{H_P}}{\mathcal{M}(a + H_P, b + a + H_P, -\lambda T_P)} \tag{S2.13}$$

We may also substitute this form of the posterior in Eq. S2.12 and Eq. S2.9 to Eq. 3, so the full model can be expressed as:

$$P(H_F > 0|\bullet, H_P) = 1 - \int_0^1 \pi(\phi|\lambda, T_P, H_P) \cdot P(H_F = 0|c\lambda T_F, \phi) d\phi \tag{S2.14}$$

where $\bullet$ represents the model parameters.

In the case that the prior follows a beta distribution, we can use the analytic form of the posterior distribution, and find that this expression can be simplified further using the confluent hypergeometric function such that

$$P(H_F > 0|\bullet, H_P) = 1 - \int_0^1 \frac{\pi(\phi) \cdot e^{-\phi\lambda T_P} \phi^{H_P}}{\mathcal{M}(a + H_P, b + a + H_P, -\lambda T_P)} \cdot e^{-\phi c\lambda t_F} d\phi \tag{S2.15}$$

$$= 1 - \frac{\int_0^1 \pi(\phi) \cdot e^{-\phi\lambda(T_P + cT_F)} \phi^{H_P} d\phi}{\mathcal{M}(a + H_P, b + a + H_P, -\lambda T_P)} \tag{S2.16}$$

$$= 1 - \frac{\mathcal{M}(a + H_P, b + a + H_P, -\lambda(T_P + cT_F))}{\mathcal{M}(a + H_P, b + a + H_P, -\lambda T_P)} \tag{S2.17}$$

Additionally we may instead define $\pi(\phi)$ as a mixture of K beta distributions such that

$$\pi(\phi) \sim \sum_{k=1}^{K} \omega_k \cdot \text{Beta}(a_k, b_k) \tag{S2.18}$$

$$\sum_{k=1}^{K} \omega_k = 1 \tag{S2.19}$$

Following a similar set of steps to those described for the single beta distribution, we

derive

$$P(H_F > 0|\bullet, H_P) = 1 - \frac{\sum_{k=1}^{K} \omega_k C_k \mathcal{M}(a_k + H_P, b_k + a_k + H_P, -\lambda(T_P + T_F))}{\sum_{k=1}^{K} \omega_k C_k \mathcal{M}(a_k + H_P, b_k + a_k + H_P, -\lambda T_P)} \tag{S2.20}$$

$$C_k = \frac{\Gamma(a_k + H_P)\Gamma(a_k + b_k)}{\Gamma(a_k)\Gamma(b_k + a_k + H_P)} \tag{S2.21}$$

Therefore, we have derived a solution to the model when the number of past and future spillover events are defined by Poisson distributions with fixed rates. This addition to the model also allows for cases where there have been host jumps in the past.

References

1. Abramowitz M, Stegun IA. Handbook of mathematical functions with formulas, graphs, and mathematical tables. vol. 55. US Government printing office; 1968.
