# Supplementary material for: Pathogen host jump risk is not predicted by spillover rate, but rather by novelty
Source: PLoS Biol. 2026 Mar 19;24(3):e3003640. doi: 10.1371/journal.pbio.3003640 (PMC13001934; doi:10.1371/journal.pbio.3003640)
Supplement: S3 Text — (PDF) [file pbio.3003640.s003.pdf]

S3 Text. Count model

First, we define our prior using a beta distribution, such that $\pi(\phi) \sim \text{Beta}(a,\ b)$. We make the same assumptions as in the main text that the outcome of each spillover event is independent of all others, and that ϕ is not changing over time. Under these assumptions, we can use a binomial distribution to quantify the probability that exactly H_P host jumps occured in N past spillover events, and each spillover had some fixed probability ϕ of resulting in a host jump. This yields:

$$P(H_P|N,\phi) = \binom{N}{H_P}\phi^{H_P}(1-\phi)^{N-H_P} \tag{S3.1}$$

Using Bayes' theorem, we can update $\pi(\phi)$ using the outcomes of these past spillover events, where the posterior distribution has the form

$$\pi(\phi|H_P,N) = \frac{\pi(\phi)\cdot P(H_P|N,\phi)}{\int_0^1 \pi(\phi)\cdot P(H_P|N,\phi)d\phi} \tag{S3.2}$$

Using the fact that the beta and binomial distributions have a conjugate relationship, it is known that the posterior distribution will follow a beta distribution, parameterized such that

$$\pi(\phi|H_P,N) \sim \text{Beta}(a+H_P,\ b+N-H_P) \tag{S3.3}$$

We then use the posterior distribution in Eq. S3.3 to compute the probability of at least one future host jump ($H_F > 0$) in some number of future spillover events (M). We express this instead as one minus the probability that no host jumps occur in M spillover events. The probability of no host jumps in M future spillovers can be written as the product of the posterior distribution from Eq. S3.3 and Eq. S3.1 with M in place of N and H_F in place of H_P. We account for our posterior uncertainty on the value of ϕ by integrating over $[0,1]$, which encompasses all possible values of ϕ. This gives

$$P(H_F > 0|\bullet,H_P) = 1 - \int_0^1 \pi(\phi|H_P,N)\cdot P(H_F=0|M,\phi)d\phi \tag{S3.4}$$

where $\bullet$ represents the model parameters.

If we substitute in the posterior and the right-hand side of Eq. S3.1, we see that the integrand is proportional to the PDF of a beta distribution, so the integral in S3.4 can be computed analytically, yielding:

$$P(H_F > 0|\bullet,H_P) = 1 - \frac{\Gamma(b+N+M-H_P)\cdot\Gamma(a+b+N)}{\Gamma(a+b+N+M)\cdot\Gamma(b+N-H_P)} \tag{S3.5}$$

As before, we can instead define a prior distribution as a mixture of K independent beta distributions where

$$\pi(\phi) \sim \sum_{k=1}^{K}\omega_k\cdot\text{Beta}(a_k,b_k) \tag{S3.6}$$

$$\sum_{k=1}^{K}\omega_k = 1 \tag{S3.7}$$

Again using the conjugate relationship between the beta and binomial distributions following the same sequence of steps as before, we derive

$$P(H_F > 0|\bullet, H_P) = 1 - \sum_{k=1}^{K} \frac{\omega_k}{C(N)} \frac{\Gamma(a_k + b_k)}{\Gamma(a_k)\Gamma(b_k)} \frac{\Gamma(b_k + N + M - H_P) \cdot \Gamma(a_k + H_P)}{\Gamma(a_k + b_k + N + M)} \tag{S3.8}$$

where

$$C(N) = \sum_{k=1}^{K} \omega_k \frac{\Gamma(a_k + b_k)}{\Gamma(a_k)\Gamma(b_k)} \frac{\Gamma(a_k + H_P)\Gamma(b_k + N - H_P)}{\Gamma(a_k + b_k + N)} \tag{S3.9}$$

Therefore, we have closed form analytical solutions to our model when our prior on ϕ follows a beta distribution or mixture of betas, even in cases where past host jumps occured ($H_P > 0$). As a result, our model is not restricted to groups of pathogens that have not previously host jumped, and this logic could be similarly applied to cases in which public health responses might have prevented a successful host jump. More importantly, this allows us to evaluate host jump risk as a function of past spillovers, past host jumps, and future host jumps when ϕ follows a beta distribution without the need to rely on numerical integration or simulations.

Analytical solution to the limit (Count model)

Using the analytical solutions to our model that uses counts of past and future spillover events (Eqs. S3.5 and S3.8), we can now derive an analog to Eq. 5 in the main text, which gives the probability of a host jump as a pathogen's rate of spillover approaches infinity. Here, we consider how the probability of a host jump changes as the number of past spillover events (N) and future spillover events (M) become infinitely large. In the main text, we assume that the rates of past and future spillovers are linearly correlated, which is analogous to assuming that the number of past and future spillover events are linearly correlated (i.e., $M = cN$). While we focus on a linear relationship, we also consider how a non-linear relationship would affect the value of the limit, where we define the number of future spillover events (M) as a differentiable function of past spillover events (N) such that $M = f(N)$. As in the main text, for this derivation, we assume no host jumps have occured in the past (i.e., $H_P = 0$) but this need not be the case in general. Substituting this into Eq. S3.5 with $H_P = 0$ and taking the limit as N approaches infinity, we get:

$$\lim_{N\to\infty} \left(1 - \frac{\Gamma(b + N + M) \cdot \Gamma(a + b + N)}{\Gamma(a + b + N + M) \cdot \Gamma(b + N)}\right) \tag{S3.10}$$

$$= 1 - \lim_{N\to\infty} \left(\frac{\Gamma(b + f(N) + N) \cdot \Gamma(a + b + N)}{\Gamma(a + b + f(N) + N) \cdot \Gamma(b + N)}\right) \tag{S3.11}$$

In the case when $f(N) = cN$ and parameters a, b, and c are all positive, non-zero integers, we can rewrite this expression using the relationship between the gamma function and factorial expressions, where $(N - 1)! = \Gamma(N)$. Using this relationship and simplifying the argument of the limit in Eq. S3.11:

$$\frac{\Gamma(b+(c+1)N)\cdot\Gamma(a+b+N)}{\Gamma(a+b+(c+1)N)\cdot\Gamma(b+N)} = \frac{(b+(c+1)N-1)!\cdot(a+b+N-1)!}{(a+b+(c+1)N-1)!\cdot(b+N-1)!} \tag{S3.12}$$

$$= \frac{(b+(c+1)N-1)!}{(a+b+(c+1)N-1)!}\frac{(a+b+N-1)!}{(b+N-1)!} \tag{S3.13}$$

$$= \frac{\prod_{k=1}^{a} a+b+N-k}{\prod_{k=1}^{a} a+b+(c+1)N-k} \tag{S3.14}$$

$$= \prod_{k=1}^{a} \frac{a+b+N-k}{a+b+(c+1)N-k} \tag{S3.15}$$

Substituting this simplified form back into Eq. S3.11,

$$1 - \lim_{N\to\infty} \frac{\Gamma(b+(c+1)N)\cdot\Gamma(a+b+N)}{\Gamma(a+b+(c+1)N)\cdot\Gamma(b+N)} = 1 - \lim_{N\to\infty} \prod_{k=1}^{a} \frac{a+b+N-k}{a+b+(c+1)N-k} \tag{S3.16}$$

Using properties of limits, the limit of a product is equal to the product of the limit for each term individually, given that each limit exists. Applying this property, we see that the limit of each term in the product does exist. Thus we can evaluate the limit and simplify, yielding

$$1 - \lim_{N\to\infty} \prod_{k=1}^{a} \frac{a+b+N-k}{a+b+(c+1)N-k} = 1 - \prod_{k=1}^{a} \lim_{N\to\infty} \frac{N+a+b-k}{(c+1)N+a+b-k} \tag{S3.17}$$

$$= 1 - \prod_{k=1}^{a} \frac{1}{(c+1)} \tag{S3.18}$$

$$= 1 - \left(\frac{1}{(c+1)}\right)^{a} \tag{S3.19}$$

Indeed, the probability of a host jump approaches a value between zero and one. However, this proof is only valid if parameters a, b, and c are all restricted to positive non-zero integer values, and we would like to prove that this claim holds for all valid parameter values. Since there are no straightforward simplifying steps when a, b, and c are not integers, we use Stirling's approximation for the gamma function, which converges asymptotically to the gamma function as N approaches infinity. For now, we focus just on the limit term from Eq. S3.11, and applying Stirling's approximation yields:

$$\lim_{N\to\infty} \frac{\sqrt{2\pi(b+f(N)+N-1)}\left(\frac{b+f(N)+N-1}{e}\right)^{b+f(N)+N-1}}{\sqrt{2\pi(a+b+f(N)+N-1)}\left(\frac{a+b+f(N)+N-1}{e}\right)^{a+b+f(N)+N-1}} \cdot \frac{\sqrt{2\pi(a+b+N-1)}\left(\frac{a+b+N-1}{e}\right)^{a+b+N-1}}{\sqrt{2\pi(b+N-1)}\left(\frac{b+N-1}{e}\right)^{b+N-1}} \tag{S3.20}$$

To simplify this expression, we define the placeholder variables

$$A = b+f(N)+N-1 \tag{S3.21}$$

$$B = a+b+N-1 \tag{S3.22}$$

$$C = a+b+f(N)+N-1 \tag{S3.23}$$

$$D = b+N-1 \tag{S3.24}$$

such that Eq. S3.20 can be written as:

$$\lim_{N\to\infty} \sqrt{\frac{A \cdot B}{C \cdot D}} \cdot \frac{e^{C+D}}{e^{A+B}} \cdot \left(\frac{A^A B^B}{C^C D^D}\right) \tag{S3.25}$$

If we assume that the limit exists for each of these three terms, this limit can be expressed as the product of the limit of each term individually. We compute the limit for the first term in Eq. S3.25 for any functional relationship between past and future spillover such that

$$\mathcal{L}_1 = \lim_{N\to\infty} \sqrt{\frac{A \cdot B}{C \cdot D}} \tag{S3.26}$$

$$= \lim_{N\to\infty} \sqrt{\frac{(b + f(N) + N - 1) \cdot (a + b + N - 1)}{(a + b + f(N) + N - 1) \cdot (b + N - 1)}} \tag{S3.27}$$

$$= \lim_{N\to\infty} \sqrt{\frac{f(N) \cdot N + N^2 + l.o.t.}{f(N) \cdot N + N^2 + l.o.t.}} \tag{S3.28}$$

$$\mathcal{L}_1 = 1, \tag{S3.29}$$

where "$l.o.t$" represents lower order terms.Regardless of the functional relationship $f(N)$, both the numerator and denominator will grow at the same rate as N approaches infinity, and this limit will always approach a constant value of one. Since we have shown that $\mathcal{L}_1$ exists, we then compute the limit of the second term of Eq. S3.25.

$$\mathcal{L}_2 = \lim_{N\to\infty} \frac{e^{C+D}}{e^{A+B}} \tag{S3.30}$$

$$= \lim_{N\to\infty} \frac{e^{a+b+f(N)+N+b+N-2}}{e^{b+f(N)+N+a+b+N-2}} \tag{S3.31}$$

$$= \lim_{N\to\infty} \frac{e^{f(N)+2N+a+b+b-2}}{e^{f(N)+2N+b+a+b-2}} \tag{S3.32}$$

$$\mathcal{L}_2 = 1 \tag{S3.33}$$

We see that the numerator and denominator will always be exactly equal for any choice of $f(N)$, and this limit will also always evaluate to one. Again, because we have shown that $\mathcal{L}_2$ exists, we then compute the limit of the third term of Eq. S3.25.

$$\mathcal{L}_3 = \lim_{N\to\infty} \left(\frac{A^A B^B}{C^C D^D}\right) \tag{S3.34}$$

$$= \lim_{N\to\infty} \left(\frac{(b+f(N)+N-1)^{(b+f(N)+N-1)}(a+b+N-1)^{(a+b+N-1)}}{(a+b+f(N)+N-1)^{(a+b+f(N)+N-1)}(b+N-1)^{b+N-1}}\right) \tag{S3.35}$$

$$= \lim_{N\to\infty} \left(\frac{(b+f(N)+N-1)^{(b+f(N)+N-1)}(a+b+N-1)^{(a+b+N-1)}}{(a+b+f(N)+N-1)^{(a+b+f(N)+N-1)}(b+N-1)^{b+N-1}}\right) \cdot \frac{(b+N-1)^a}{(b+N-1)^a} \cdot \frac{(b+f(N)+N-1)^a}{(b+f(N)+N-1)^a} \tag{S3.36}$$

$$= \lim_{N\to\infty} \left(\frac{b+f(N)+N-1}{a+b+f(N)+N-1}\right)^{a+b+f(N)+N-1} \cdot \left(\frac{a+b+N-1}{b+N-1}\right)^{a+b+N-1} \cdot \left(\frac{b+N-1}{b+f(N)+N-1}\right)^{a} \tag{S3.37}$$

We simplify this expression to a product of three terms. Again, we assume the limit exists for each of the terms in Eq. S3.37 and evaluate each of these limits individually. For the first term, we get

$$l_1 = \lim_{N\to\infty} \left(\frac{b+f(N)+N-1}{a+b+f(N)+N-1}\right)^{a+b+f(N)+N-1} \tag{S3.38}$$

$$ln(l_1) = \lim_{N\to\infty} (a+b+f(N)+N-1) \cdot ln\left(\frac{b+f(N)+N-1}{a+b+f(N)+N-1}\right) \tag{S3.39}$$

$$= \lim_{N\to\infty} \frac{ln\left(\frac{b+f(N)+N-1}{a+b+f(N)+N-1}\right)}{\frac{1}{(a+b+f(N)+N-1)}} = \frac{0}{0} \tag{S3.40}$$

applying L'Hopitals rule and taking the derivatives of the numerator and denominator:

$$= \lim_{N\to\infty} \frac{\frac{f'(N)+1}{b+f(N)+N-1} - \frac{f'(N)+1}{a+b+f(N)+N-1}}{\frac{-(f'(N)+1)}{(a+b+f(N)+N-1)^2}} \tag{S3.41}$$

$$= \lim_{N\to\infty} \frac{\frac{1}{b+f(N)+N-1} - \frac{1}{a+b+f(N)+N-1}}{\frac{-1}{(a+b+f(N)+N-1)^2}} \tag{S3.42}$$

$$= \lim_{N\to\infty} \frac{-a\cdot(a+b+f(N)+N-1)^2}{(a+b+f(N)+N-1)(b+f(N)+N-1)} \tag{S3.43}$$

$$= -a \lim_{N\to\infty} \frac{a+b+f(N)+N-1}{(b+f(N)+N-1)} = -a \tag{S3.44}$$

$$ln(l_1) = -a \tag{S3.45}$$

$$l_1 = e^{-a} \tag{S3.46}$$

Because we use L'Hopital's rule to evaluate this limit, we have an additional constraint on $f(N)$, which is that it must be differentiable. If this condition is met, however, we

see again that the chosen functional relationship between past and future spillover does not change the value of this limit. Again, this is because the chosen functional form appears in the numerator and denominator of the limit argument, so the rates of growth would exactly balance. Since l_1 exists, we use a similar sequence of steps to evaluate the limit of the second term in Eq. S3.37 such that

$$l_2 = \lim_{N\to\infty} \left(\frac{a+b+N-1}{b+N-1}\right)^{a+b+N-1} \tag{S3.47}$$

$$ln(l_2) = \lim_{N\to\infty} (a+b+N-1)\cdot ln\left(\frac{a+b+N-1}{b+N-1}\right) \tag{S3.48}$$

$$= \lim_{N\to\infty} \frac{ln\left(\frac{a+b+N-1}{b+N-1}\right)}{\frac{1}{(a+b+N-1)}} = \frac{0}{0} \tag{S3.49}$$

Applying L'Hopitals rule and taking the derivatives of the numerator and denominator:

$$= \lim_{N\to\infty} \frac{\frac{1}{b+N-1} - \frac{1}{a+b+N-1}}{\frac{-1}{(a+b+N-1)^2}} \tag{S3.50}$$

$$= \lim_{N\to\infty} \frac{a\cdot(a+b+N-1)^2}{(a+b+N-1)(b+N-1)} \tag{S3.51}$$

$$= a \lim_{N\to\infty} \frac{a+b+N-1}{(b+N-1)} = a \tag{S3.52}$$

$$ln(l_2) = a \tag{S3.53}$$

$$l_2 = e^a \tag{S3.54}$$

The functional relationship between past and future spillover $f(N)$ is not present in this term, so alternative functional relationships would not affect the value of this limit. Since l_2 exists, we use a similar sequence of steps to evaluate the limit of the third term in Eq. S3.37 such that

$$l_3 = \lim_{N\to\infty} \left(\frac{b+N-1}{b+f(N)+N-1}\right)^a \tag{S3.55}$$

$$= \left(\lim_{N\to\infty} \frac{b+N-1}{b+f(N)+N-1}\right)^a \tag{S3.56}$$

Here, we see that the functional relationship between past and future spillover would affect the value of this limit. When the function $f(N)$ grows at a rate that is greater than linear, the limit evaluates to zero, and if $f(N)$ grows at a rate that is less than linear, the limit evaluates to one. We are interested in the case when the relationship between the number of past and future spillover events is exactly linear (i.e., $f(N) = cN$). If we substitute this expression into Eq. S3.56, we can evaluate the limit such that

$$l_3 = \lim_{N\to\infty} \left(\frac{b+N-1}{b+(c+1)N-1}\right)^a \tag{S3.57}$$

$$l_3 = \left(\frac{1}{(c+1)}\right)^a \tag{S3.58}$$

In any case, this limit does exist, so we can express the value of $\mathcal{L}_3$, noting that l_1 and

l_2 exactly cancel one another such that

$$\mathcal{L}_3 = l_1 \cdot l_2 \cdot l_3 \tag{S3.59}$$

$$\mathcal{L}_3 = e^a e^{-a} \cdot l_3 \tag{S3.60}$$

$$\mathcal{L}_3 = l_3 \tag{S3.61}$$

Therefore, when the number of past and future spillover events are linearly correlated, we can express $\mathcal{L}_3$ as

$$\mathcal{L}_3 = \frac{1}{(c+1)^a} \tag{S3.62}$$

Because $\mathcal{L}_1$, $\mathcal{L}_2$, and $\mathcal{L}_3$ all exist, and $\mathcal{L}_1 = \mathcal{L}_2 = 1$ for any differentiable relationship $(f(N))$, we can express the value of the limit in Eq. S3.11 under a linear relationship as:

$$P_\infty(H_F > 0|\bullet, H_P = 0) = 1 - \lim_{N\to\infty}\left(\frac{\Gamma(b + (c+1)\cdot N)\cdot\Gamma(a+b+N)}{\Gamma(a+b+(c+1)\cdot N)\cdot\Gamma(b+N)}\right) \tag{S3.63}$$

$$= 1 - \mathcal{L}_1 \cdot \mathcal{L}_2 \cdot \mathcal{L}_3 \tag{S3.64}$$

$$= 1 - \frac{1}{(c+1)^a} \tag{S3.65}$$

From this, we can also see that this limit will take a value of either zero or one when $f(N)$ is non-linear. However, when this relationship is linear, we see that this expression is identical to the value derived in the case in Eq. S3.19 when the parameters a, b, and c were restricted to positive integer values. Because Stirling's approximation converges asymptotically to the gamma function for large values, we are able to exactly compute this limit and find a general form that is also valid for non-integer parameter values.

This derivation therefore demonstrates that the probability of a host jump, assuming a linear relationship between the number of past and future spillover events, saturates to a value between zero and one, even when the rate of spillover approaches infinite values. This result arises because a large number of past spillover events without a host jump drives the posterior density of ϕ towards zero, while a large number of future spillover events yields many opportunities for a future host jump. These competing factors exactly counterbalance one another in the limit when there is a linear correlation between the rate of spillover in the past and future. However, non-linear relationships between past and future spillover do not maintain this balance and do indeed converge exactly to zero or one.

Analytical solution to the limit for a beta mixture distribution

We also consider the value of this limit when the prior follows a mixture of beta distributions. Using the analytical solution in Eq. S3.8 for $H_P = 0$, this limit can be written for any mixture of K beta distributions such that

$$P_\infty(H_F > 0|\bullet, H_P = 0) = 1 - \lim_{N\to\infty}\sum_{k=1}^{K}\frac{\omega_k}{C(N)}\frac{\Gamma(a_k+b_k)}{\Gamma(a_k)\Gamma(b_k)}\frac{\Gamma(b_k+N+M)\cdot\Gamma(a_k)}{\Gamma(a_k+b_k+N+M)} \tag{S3.66}$$

$$C(N) = \sum_{j=1}^{K}\omega_j\frac{\Gamma(a_j+b_j)}{\Gamma(a_j)\Gamma(b_j)}\frac{\Gamma(a_j)\Gamma(b_j+N)}{\Gamma(a_j+b_j+N)} \tag{S3.67}$$

To condense this equation, we use the beta function, which is defined as

$$B(a,b) = \frac{\Gamma(a)\Gamma(b)}{\Gamma(a+b)}; \;\; B^{-1}(a,b) = \frac{\Gamma(a+b)}{\Gamma(a)\Gamma(b)} \tag{S3.68}$$

Rewriting Eq. S3.67 with the beta function and substituting $M = cN$, we get

$$P_\infty(H_F > 0|\bullet, H_P = 0) = 1 - \lim_{N\to\infty} \sum_{k=1}^{K} \frac{\omega_k B^{-1}(a_k, b_k) B(a_k, B_k + (c+1)N)}{\sum_{j=1}^{K} \omega_j B^{-1}(a_j, b_j) B(a_j, b_j + N)} \quad \text{(S3.69)}$$

Using the fact that the limit of a sum is equal to the sum of the limit of each individual term, we can rewrite this as

$$P_\infty(H_F > 0|\bullet, H_P = 0) = 1 - \sum_{k=1}^{K} \lim_{N\to\infty} \frac{\omega_k B^{-1}(a_k, b_k) B(a_k, B_k + N + M)}{\sum_{j=1}^{K} \omega_j B^{-1}(a_j, b_j) B(a_j, b_j + N)} \quad \text{(S3.70)}$$

$$= 1 - \sum_{k=1}^{K} \lim_{N\to\infty} \frac{T_k(N)}{\sum_{j=1}^{K} D_j(N)} \quad \text{(S3.71)}$$

where $T_k(N)$ and $D_j(N)$ are shorthand representations of the terms in the numerator and denominator respectively. Because of the sum in the denominator, computing this limit is not straightforward, so we instead find an upper and lower bound for this limit. For a sufficiently large value of N, we want to show that there is some j^* such that $D_{j^*}(N) \geq D_j(N)$ for all values of j. Representing this using limits, we have

$$\lim_{N\to\infty} D_j(N) \leq \lim_{N\to\infty} D_{j^*}(N) \quad \text{(S3.72)}$$

$$\lim_{N\to\infty} \frac{D_j(N)}{D_{j^*}(N)} \leq 1 \quad \text{(S3.73)}$$

By substituting in the expression for $D_j(N)$ and using Stirling's approximation, we evaluate the limit on the left-hand side of Eq. S3.73 to express this inequality in terms of the parameters of the beta mixture distribution.

$$\lim_{N\to\infty} \frac{\omega_j B^{-1}(a_j, b_j) B(a_j, b_j + N)}{\omega_j B^{-1}(a_{j^*}, b_{j^*}) B(a_{j^*}, b_{j^*} + N)} \quad \text{(S3.74)}$$

$$= \frac{\omega_j \Gamma(a + b_j)\Gamma(b_{j^*})}{\omega_{j^*}\Gamma(b_j)\Gamma(a_{j^*} + b_{j^*})} \lim_{N\to\infty} \frac{\Gamma(b_j + N)\Gamma(a_{j^*} + b_{j^*} + N)}{\Gamma(a_j + b_j + N)\Gamma(b_{j^*} + N)} \quad \text{(S3.75)}$$

$$= C \cdot \lim_{N\to\infty} T_1 \cdot T_2 \cdot T_3 \quad \text{(S3.76)}$$

where

$$C = \frac{\omega_j \Gamma(a + b_j)\Gamma(b_{j^*})}{\omega_{j^*}\Gamma(b_j)\Gamma(a_{j^*} + b_{j^*})} \quad \text{(S3.77)}$$

$$T_1 = \sqrt{\frac{(b_j + N - 1)(a_{j^*} + b_{j^*} + N - 1)}{(a_j + b_j + N - 1)(b_{j^*} + N - 1)}} \quad \text{(S3.78)}$$

$$T_2 = \frac{e^{(a_j + b_j + N - 1) + (b_{j^*} + N - 1)}}{e^{(b_j + N - 1) + (a_{j^*} + b_{j^*} + N - 1)}} \quad \text{(S3.79)}$$

$$T_3 = \left(\frac{b_j + N - 1}{a_j + b_j + N - 1}\right)^{a_j + b_j + N - 1} \cdot \left(\frac{a_{j^*} + b_{j^*} + N - 1}{b_{j^*} + N - 1}\right)^{a_{j^*} + b_{j^*} + N} \cdot \frac{(b_{j^*} + N - 1)^{a_{j^*}}}{(b_j + N - 1)^{a_j}} \quad \text{(S3.80)}$$

We can evaluate the limits of T_1, T_2, T_3 using a similar approach to the steps taken in the derivation with a single beta distribution. The limits of these three terms can be

taken individually, where the steps to evaluate the limit of T_1 corresponds to the calculation of $\mathcal{L}_1$, and the same is true of T_2 and $\mathcal{L}_2$, and T_3 and $\mathcal{L}_3$ respectively. Applying these steps we get

$$\lim_{N\to\infty} T_1 = 1 \tag{S3.81}$$

$$\lim_{N\to\infty} T_2 = e^{a_j} e^{-a_{j^*}} \tag{S3.82}$$

$$\lim_{N\to\infty} T_3 = e^{-a_j} e^{a_{j^*}} \cdot t_3 \tag{S3.83}$$

$$t_3 = \lim_{N\to\infty} \frac{(b_{j^*} + N - 1)^{a_{j^*}}}{(b_j + N - 1)^{a_j}} \tag{S3.84}$$

The value of the limit in its entirety consists of the product of these terms, and therefore only depends on the values of a_j and a_{j^*} in the t_3 term. In particular, the limit is zero if $a_{j^*} < a_j$, undefined if $a_{j^*} > a_j$, and one if $a_{j^*} = a_j$. Therefore the inequality in Eq. S3.73 is always satisfied when $a_{j^*} < a_j$. Thus, for a significantly large N, we can define the index of the largest value of $D_j(N)$ such that j^* corresponds to the mixture component(s) with the smallest value of a_j. Even when there is not a unique smallest value of a_j, any value in the set of j^* would provide almost equivalent upper bounds. To ensure the tightest possible upper and lower bounds it is necessary to find the unique value in j^* that maximizes $D_j(N)$. This is given by the j^* that corresponds with the largest value of b_{j^*}. We then define this unique index (Ω) such that $D_\Omega \geq D_j$ for all values of j.

Using this observation, we define an upper and lower bound on the limit from Eq. S3.71 such that

$$\sum_{k=1}^{K} \lim_{N\to\infty} \frac{T_k(N)}{\sum_{j=1}^{K} D_\Omega(N)} \leq \sum_{k=1}^{K} \lim_{N\to\infty} \frac{T_k(N)}{\sum_{j=1}^{K} D_j(N)} \leq \sum_{k=1}^{K} \lim_{N\to\infty} \frac{T_k(N)}{D_\Omega(N)} \tag{S3.85}$$

For the upper bound term, we substitute the expressions for the numerator and denominator, so our upper bound limit can be expressed as

$$\begin{aligned} &\sum_{k=1}^{K} \lim_{N\to\infty} \frac{\omega_k B^{-1}(a_k, b_k) B(a_k, b_k + (c+1)N)}{\sum_{j=1}^{K} \omega_j B^{-1}(a_j, b_j) B(a_j, b_j + N)} \leq \\ &\sum_{k=1}^{K} \lim_{N\to\infty} \frac{\omega_k B^{-1}(a_k, b_k) B(a_k, b_k + (c+1)N)}{\omega_\Omega B^{-1}(a_\Omega, b_\Omega) B(a_\Omega, b_\Omega + N)} \end{aligned} \tag{S3.86}$$

Using the limit derivation from Eq. S3.84 and the fact that $a_\Omega \leq a_k$ for all values of k, we see that every limit term within the sum will be zero, except when $k \in j^*$, and applying these results, we can rewrite and compute the limit such that

$$\begin{aligned} &\sum_{k=1}^{K} \lim_{N\to\infty} \frac{\omega_k B^{-1}(a_k, b_k) B(a_k, b_k + (c+1)N)}{\sum_{j=1}^{K} \omega_j B^{-1}(a_j, b_j) B(a_j, b_j + N)} \leq \\ &\frac{1}{(1+c)^{a_\Omega}} \sum_{k\in j^*} \frac{\omega_k \Gamma(a_k + b_k)\Gamma(b_\Omega)}{\omega_\Omega \Gamma(a_\Omega + b_\Omega)\Gamma(b_k)} \end{aligned} \tag{S3.87}$$

The lower bound can be computed similarly, such that

$$\begin{aligned} &\frac{1}{K \cdot (1+c)^{a_\Omega}} \sum_{k\in j^*} \frac{\omega_k \Gamma(a_k + b_k)\Gamma(b_\Omega)}{\omega_\Omega \Gamma(a_\Omega + b_\Omega)\Gamma(b_k)} \leq \\ &\sum_{k=1}^{K} \lim_{N\to\infty} \frac{\omega_k B^{-1}(a_k, b_k) B(a_k, B_k + (c+1)N)}{\sum_{j=1}^{K} \omega_j B^{-1}(a_j, b_j) B(a_j, b_j + N)} \end{aligned} \tag{S3.88}$$

Thus, when there is a single smallest value of a_j, the lower and upper bounds are exactly equal. For this case, we have an exact analytical solution for the limit when the prior distribution follows a mixture of beta distributions, such that

$$P_\infty(H_F > 0|\bullet, H_P = 0) = 1 - \frac{1}{(1+c)^{a_\Omega}} \tag{S3.89}$$

When there is not a unique smallest value of a_j, the lower and upper bounds are not necessarily equal, so we can only analytically compute bounds for the limit. Since we are interested in the probability of at least one host jump in the future, we only consider the upper bound on this probability as a conservative approach. To do this, we substitute our analytically computed lower bound, which gives

$$P_\infty(H_F > 0|\bullet, H_P = 0) = 1 - \sum_{k=1}^{K} \lim_{N\to\infty} \frac{\omega_k B^{-1}(a_k, b_k) B(a_k, B_k + (c+1)N)}{\sum_{j=1}^{K} \omega_j B^{-1}(a_j, b_j) B(a_j, b_j + N)} \tag{S3.90}$$

$$P_\infty(H_F > 0|\bullet, H_P = 0) \leq 1 - \frac{1}{K \cdot (1+c)^{a_\Omega}} \sum_{k\in j^*} \frac{\omega_k \Gamma(a_k + b_k)\Gamma(b_\Omega)}{\omega_\Omega \Gamma(a_\Omega + b_\Omega)\Gamma(b_k)} \tag{S3.91}$$

The gamma functions in this expression are strictly positive because parameters ω_k, a_k, b_k, and c are all greater than zero for all values of k, every term, we notice

$$\frac{1}{K \cdot (1+c)^{a_\Omega}} \sum_{k\in j^*} \frac{\omega_k \Gamma(a_k + b_k)\Gamma(b_\Omega)}{\omega_\Omega \Gamma(a_\Omega + b_\Omega)\Gamma(b_k)} > 0 \tag{S3.92}$$

We intuitively expect that this limit is also equal to the case with a single smallest value of a_{j*} shown in Eq. S3.89, but we are unable to show this analytically. Nevertheless, we have demonstrated that, when there is a linear correlation between the number of spillover events in the past and future, the probability of a host jump saturates to a value between zero and one as the rate of spillover approaches infinite values, and this is valid for any mixture of beta distributions.
