# Supplementary material for: Pathogen host jump risk is not predicted by spillover rate, but rather by novelty
Source: PLoS Biol. 2026 Mar 19;24(3):e3003640. doi: 10.1371/journal.pbio.3003640 (PMC13001934; doi:10.1371/journal.pbio.3003640)
Supplement: S4 Text — (PDF) [file pbio.3003640.s004.pdf]

S4 Text. Model validation using simulations and count parameterization

To validate the analytical solution to our model, we use a stochastic model to simulate the probability of a future host jump ($H_F > 0$) for a given rate of spillover for the past (λ) and future ($c\lambda$), where there were exactly H_P host jumps in the past. First, we simulate a random value of ϕ from our prior distribution $\pi(\phi)$, which we call ϕ_r. We then use a Poisson distribution with mean λT_P to determine the number of past spillover events (N). Then, to determine the number of past host jumps (H_P), we use a binomial distribution with parameters ($n = N,\ p = \phi_r$), so each spillover had probability ϕ_r to result in a host jump. If this random value is exactly equal to the specified number of past host jumps ($H = H_P$), we store this value of ϕ_r. Otherwise ($H \neq H_P$), the value of ϕ_r is discarded. We then simulate a new value of ϕ_r and repeat this process until we have stored 50,000 values of ϕ_r.

We then simulate the number of future host jumps (H_F) using a similar set of steps. We determine the number of future spillover events (M) using a Poisson distribution with mean $c\lambda T_F$. We then use a binomial distribution with parameters ($n = M,\ p = \phi_r$), to determine if a host jump will occur in M future spillover events. We repeat this process with a new simulated value of M for every stored value of ϕ_r. The fraction of these binomial draws that resulted in at least one host jump ($H_F > 0$) approximates the probability of a host jump given the model parameters $\lambda T_P,\ c\lambda T_F,\ H_P$, and $\pi(\phi)$. In Fig. S4.1 we see that the results of these simulations do indeed align with our analytical solution and produce the same qualitative trends suggested by our main results.

Additionally, we present an alternative visualization of our main conclusion that the length of historical association is predictive of host jump risk but that spillover rate is not. In Fig. S4.2, we evaluate our model for different hypothetical pathogen types that differ in either their spillover rate or the size of their past spillover window. We then calculate the probability of a host jump for each in a future spillover window of $T_F = 5$ years. The three scenarios (prior distributions) used here are the same as those used in the main text (see Fig. 2 A-C). We see that, within each scenario, the riskiest pathogens are those with a shorter duration of historical association (i.e., smaller T_P). Furthermore, we see that, depending on the scenario, the riskier pathogen may be the one that spills over at the lower rate or higher rate. This illustrates that spillover rate is not predictive of host jump risk, particularly when we cannot definitively quantify our uncertainty in the probability that a given spillover event will result in a host jump (ϕ).

We also use our analytical solution for the count model derived in Supporting information S3 Text, with results shown in Fig. S4.3. We also validated these results using the simulation in Fig. S4.4, and we see that the simulation produces essentially the same results as our analytical solution for the count-based model. Because time is implicit in the count model, it is not possible to replicate Fig. 2J-L, but we see that this model still yields qualitatively similar results to the model presented in the main text, wherein the riskiest pathogens may be those that spill over at high, low, or intermediate rates.

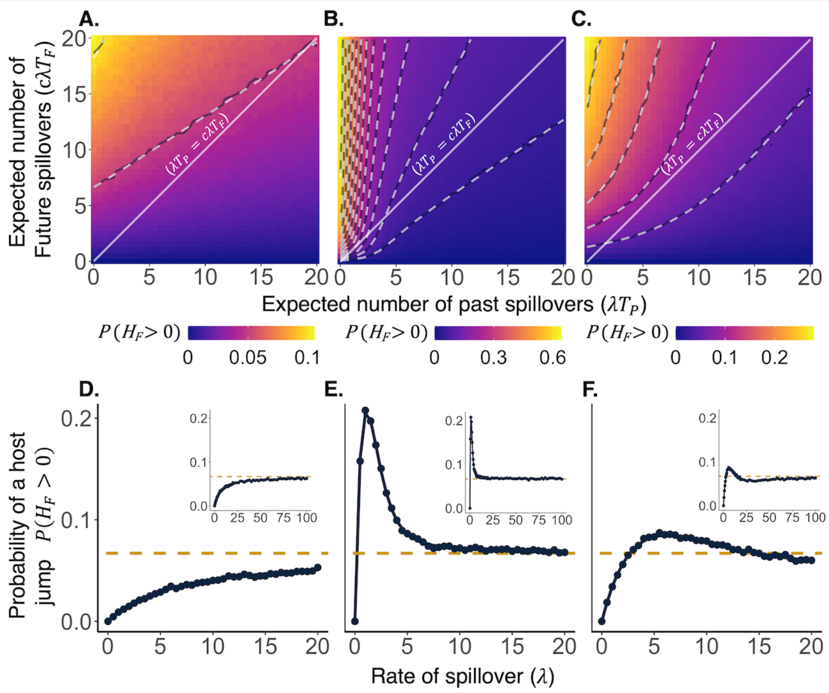

Fig S4.1. Simulation results of the Poisson model align with the analytical model solution. When the number of spillover events in the past and future follow independent Poisson distributions, our model results are qualitatively similar to the results found in the main text. Panels A-C show the probability of a future host jump for each of our three scenarios from the main text when the number of past and future spillover events follow Poisson distributions with fixed rates λ and $c\lambda$ respectively. We generate these probabilities using 50,000 replicates in our simulation, and the contour lines are shown for every 5% increment of host jump risk for the simulation (solid line) and our derived numerical solution (dashed line). In panels D-F, we show host jump risk when past and future rate parameters are linearly correlated for both the simulation (points) and our derived numerical solution (solid line). These trends are qualitatively similar to the results shown in the main text. It appears that very high rates of spillover may approach the same value derived in Supporting information S5 Text (dashed gold line). For each panel, the remaining parameter values are $T_P = 1$, $T_F = 1$, $c = 1$. The code needed to generate this figure can be found in https://doi.org/10.5281/zenodo.14154724

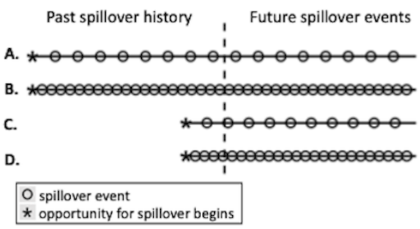

Future host jump probability			
	Scenario 1	Scenario 2	Scenario 3
A.	0.018	0.030	0.027
B.	0.027	0.029	0.027
C.	0.035	0.243	0.121
D.	0.095	0.121	0.100

Long past history (A, B); Short past history (C, D)

low risk — high risk

Fig S4.2. Length of historical association, not spillover rate, is predictive of host jump risk. We show four possible combinations of spillover-related factors that could affect host jump risk prediction, namely, spillover rate λ and the past spillover window T_P. Rows A-D show illustrative representations of past and future spillover histories, where pathogens can spill over at high (B and D) or low rates (A and C) and can have long (A and B) or short (C and D) past spillover windows with the novel host. We can then use our model to quantify the probability of a host jump in each of these four cases using the same three scenarios (prior distributions) as the main text (see Fig. 2, A-C). In each of the three scenarios, we see that the riskiest pathogens are always those with short past histories. Furthermore, we see that the riskiest pathogen may spill over at a high or low rate depending on the scenario, illustrating that spillover rate is not predictive of host jump risk. Additionally, we see that the pathogens with long past histories do not differ substantially in terms of host jump risk, despite differences in their rate of spillover. Model parameters: $\lambda = 1$ or 10, $c = 1$, $T_P = 1$ or 10, $T_F = 5$ The code needed to generate this figure and table of values can be found in https://doi.org/10.5281/zenodo.14154724

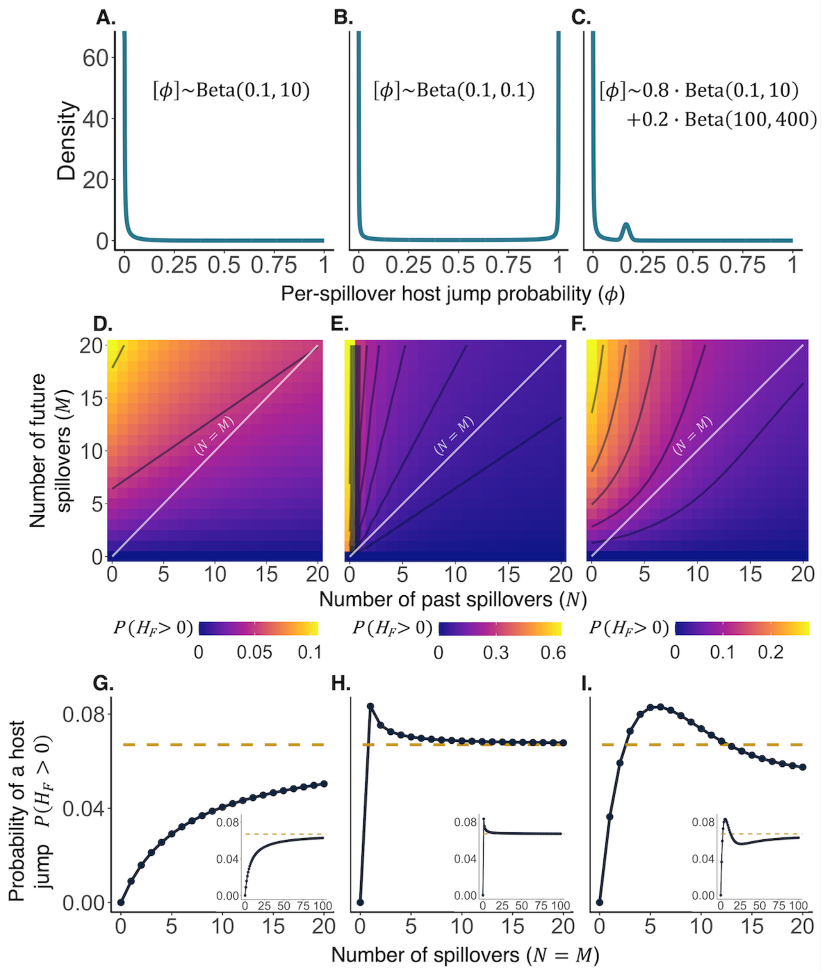

Fig S4.3. Analytical model results using counts of spillover events. The columns of panels from left to right denote Scenarios 1-3 from the main text respectively. Panels A-C show the prior distributions on ϕ. Panels D-F show the model-calculated probability of a successful host jump as a function of the number of past and future spillover events. Host jump risk increases with future spillovers (M) and decreases with past spillover (N). The contour lines in each panel demarcate changes of 5% in total host jump risk. Note that the color range differs for each panel. The solid white lines represent the 1:1 relationship between the number of past and future spillovers, and panels G-I show the probabilities of a future host jump along these lines. Comparing panels G-I reveals that depending on the prior distribution, host jump risk may increase or decrease as a function of spillover rate. Nevertheless, in all scenarios, host jump risk converges to the value $\left(1 - \frac{1}{(c+1)^a}\right)$, as spillover events increase (inset figures). The code needed to generate this figure can be found in https://doi.org/10.5281/zenodo.14154724

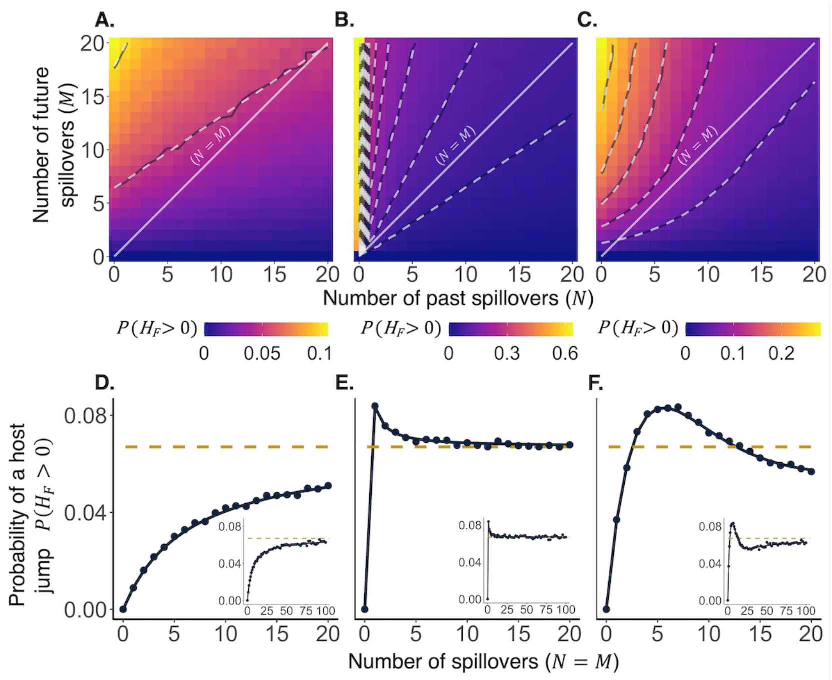

Fig S4.4. Simulation results of the count model align with the analytical model solution. Columns indicate scenarios 1-3 from the main text respectively. Panels A-C indicate the fraction of 50,000 simulations where a host jump occured for the given number of past (N) and future (M) spillover events. Contour lines representing a 5% change in host jump risk are shown for the simulation values (solid lines), which closely follow the analytic solution (dashed lines). Panels D-F show the analytical solution from the main text (line) with simulation results (points) when the number of past and future spillover events are linearly correlated. This figure confirms the results in the main text. The code needed to generate this figure can be found in https://doi.org/10.5281/zenodo.14154724
