# Supplementary material for: Pathogen host jump risk is not predicted by spillover rate, but rather by novelty
Source: PLoS Biol. 2026 Mar 19;24(3):e3003640. doi: 10.1371/journal.pbio.3003640 (PMC13001934; doi:10.1371/journal.pbio.3003640)
Supplement: S5 Text — (PDF) [file pbio.3003640.s005.pdf]

S5 Text. Analytical solution to the limit (Poisson model)

Here, we consider how increasing the rate of spillover relates to host jump risk, specifically when the rate of spillover approaches infinitely high values. Intuitively, one might expect that infinite spillover would always result in a host jump unless the probability of host jump given spillover were exactly 0. However, in the main text, we state that host jump risk converges to a value between zero and one at high rates of spillover. Here, we compute the limit of our analytical solution as spillover rates become infinitely high to validate this statement.

Using the analytical solution to our model for the case with a fixed rate of spillover over some past and future time intervals, we can take the limit as λ approaches infinity. As in the main text, we use λ to represent the rate of past spillover and $f(\lambda)$ to represent the rate of future spillover. In the main text, we assumed a linear relationship such that $f(\lambda) = c\lambda$, but we relax this assumption here to demonstrate the potential effects of non-linear relationships as well. We can then express the probability of a host jump as the rate of spillover becomes infinitely high as

$$P_\infty(H_F > 0|\bullet, H_P = 0) = \lim_{\lambda\to\infty} 1 - \frac{\mathcal{M}\left(a, a+b, -(f(\lambda)\cdot T_F + \lambda T_P)\right)}{\mathcal{M}\left(a, a+b, -\lambda T_P\right)} \tag{S5.1}$$

where $\bullet$ represents the model parameters, as in the main text. Because all of our model parameters are positive real numbers, we can apply the asymptotic behavior of the confluent hypergeometric function as the parameter $|z|$ approaches infinity [1]. Namely,

$$\mathcal{M}\left(x, y, z\right) \sim \frac{\Gamma(y)}{\Gamma(y-x)}(-z)^{-x} \tag{S5.2}$$

We can then express the limit in terms of this asymptotic form and simplify so

$$\lim_{\lambda\to\infty} 1 - \frac{\mathcal{M}\left(a, a+b, -(f(\lambda)T_F + \lambda T_P)\right)}{\mathcal{M}\left(a, a+b, -\lambda T_P\right)} = \lim_{\lambda\to\infty} 1 - \frac{\frac{\Gamma(a+b)}{\Gamma(b)}((f(\lambda)T_F + \lambda T_P))^{-a}}{\frac{\Gamma(a+b)}{\Gamma(b)}(\lambda T_P)^{-a}} \tag{S5.3}$$

$$= \lim_{\lambda\to\infty} 1 - \frac{(\lambda T_P)^a}{(f(\lambda)T_F + \lambda T_P)^a} \tag{S5.4}$$

Using this property, we can express the probability of at least one host jump as the rate of spillover becomes infinitely large as

$$P_\infty(H_F > 0|\bullet, H_P = 0) = 1 - \lim_{\lambda\to\infty} \frac{(\lambda T_P)^a}{(f(\lambda)T_F + \lambda T_P)^a} \tag{S5.5}$$

$$= 1 - \lim_{\lambda\to\infty} \frac{1}{\left(\frac{f(\lambda)}{\lambda}\cdot\frac{T_F}{T_P} + 1\right)^a} \tag{S5.6}$$

Here, we notice that the functional relationship between past and future spillover rates determines whether the probability of a future host jump approaches zero, one, or an intermediate value. Namely, if $f(\lambda)$ grows at a rate that is greater than linear (e.g., $f(\lambda = \lambda^x$ where $x > 1)$, the limit equals zero (and thus the probability of a future host jump equals one). In contrast, if $f(\lambda)$ grows at a rate that is less than linear (e.g., $f(\lambda = \lambda^x$ where $x < 1)$, the limit equals one (so the probability of a future host jump is zero). However, under a linear relationship (e.g. $f(\lambda) = c\lambda$), this equation simplifies

such that

$$P_\infty(H_F > 0|\bullet, H_P = 0) = 1 - \frac{1}{\left(c\frac{T_F}{T_P} + 1\right)^a} \lim_{\lambda\to\infty} \frac{\lambda^a}{\lambda^a} \tag{S5.7}$$

$$= 1 - \frac{1}{\left(c\frac{T_F}{T_P} + 1\right)^a} \tag{S5.8}$$

where c is the ratio between past and future spillover rates, T_P and T_F are the past and future spillover windows respectively, and a is the first shape parameter in the beta prior, $\pi(\phi)$.

Analytical solution to the limit for a beta mixture distribution (Poisson model)

We also consider the value of this limit when the prior follows a mixture of beta distributions. Using the analytical solution for a beta mixture prior derived in Supporting information S2 Text when the number of host jumps $H_P = 0$, this limit can be written for any mixture of K beta distributions such that

$$P_\infty(H_F > 0|\bullet, H_P = 0) = 1 - \lim_{\lambda\to\infty} \sum_{k=1}^{K} \frac{\omega_k}{C(\lambda)} \mathcal{M}(a, a+b, -(f(\lambda)\cdot T_F + \lambda T_P)) \tag{S5.9}$$

$$C(\lambda) = \sum_{j=1}^{K} \omega_j \mathcal{M}(a, a+b, -\lambda T_P) \tag{S5.10}$$

Where as before, we use λ to represent the rate of past spillover, we define the rate of future spillover as $f(\lambda)$, and $\bullet$ represents the model parameters. In what follows, we will assume that $f(\lambda) = c\lambda$, as our previous derivations suggest nonlinear relationships would still result in convergence to a value of zero or one depending on the direction of the relationship. Rewriting Eq. S5.10 under this linear relationship gives

$$P_\infty(H_F > 0|\bullet, H_P = 0) = 1 - \lim_{\lambda\to\infty} \sum_{k=1}^{K} \frac{\omega_k \mathcal{M}(a_k, a_k + b_k, -\lambda(cT_F + T_P))}{\sum_{j=1}^{K} \omega_j \mathcal{M}(a_j, a_j + b_j, -\lambda T_P)} \tag{S5.11}$$

Using the fact that the limit of a sum is equal to the sum of the limit of each individual term, we can rewrite this as

$$P_\infty(H_F > 0|\bullet, H_P = 0) = 1 - \sum_{k=1}^{K} \lim_{\lambda\to\infty} \frac{\omega_k \mathcal{M}(a_k, a_k + b_k, -\lambda(cT_F + T_P))}{\sum_{j=1}^{K} \omega_j \mathcal{M}(a_j, a_j + b_j, -\lambda T_P)} \tag{S5.12}$$

$$= 1 - \sum_{k=1}^{K} \lim_{\lambda\to\infty} \frac{\omega_k T_k(\lambda(cT_F + T_P))}{\sum_{j=1}^{K} \omega_j D_j(\lambda T_P)} \tag{S5.13}$$

where $T_k(\lambda)$ and $D_j(\lambda)$ are shorthand representations of the terms in the numerator and denominator respectively. Because of the sum in the denominator, computing this limit is not straightforward, so we instead find an upper and lower bound for this limit. For a sufficiently large value of λ, we want to show that that there is some j^* such that $D_{j^*}(\lambda) \geq D_j(\lambda)$ for all values of j. Representing this using limits, we have

$$\lim_{\lambda\to\infty} D_j(\lambda) \leq \lim_{\lambda\to\infty} D_{j^*}(\lambda) \tag{S5.14}$$

$$\lim_{\lambda\to\infty} \frac{D_j(\lambda)}{D_{j^*}(\lambda)} \leq 1 \tag{S5.15}$$

By substituting in the expression for $D_j(\lambda)$ and using the asymptotic relationship for the confluent hypergeometric function in Eq. S5.2, we evaluate the limit on the left-hand side of Eq. S5.15 to express this inequality in terms of the parameters of the beta mixture distribution.

$$\lim_{\lambda\to\infty} \frac{\omega_j \mathcal{M}(a_j, a_j + b_j, -\lambda)}{\omega_{j^*}\mathcal{M}(a_{j^*}, a_{j^*} + b_{j^*}, -\lambda)} \tag{S5.16}$$

$$= \frac{\omega_j \Gamma(b_j)\Gamma(a_{j^*} + b_{j^*})}{\omega_{j^*}\Gamma(b_{j^*})\Gamma(a_j + b_j)} \lim_{\lambda\to\infty} \frac{\lambda^{-a_j}}{\lambda^{-a_{j^*}}} \tag{S5.17}$$

$$= C \cdot \lim_{\lambda\to\infty} \lambda^{a_{j^*} - a_j} \tag{S5.18}$$

From this, we see that the limit does not exist when $a_{j^*} > a_j$, is equal to C when $a_{j^*} = a_j$, and zero when $a_{j^*} < a_j$. We see that the inequality in Eq. S5.15 is always satisfied when $a_{j^*} < a_j$. Thus, for a significantly large λ, we can define the index of the largest value of $D_j(\lambda)$ as j^* which gives the index of the mixture component(s) with the smallest value of a_j. Even when there is not a unique smallest value of a_j, any value in the set of j^*s would provide almost equivalent upper bounds. To ensure the tightest possible upper and lower bounds it is necessary to find the unique value in j^* that maximizes $D_j(\lambda)$. This can be determined a priori, as this value only depends on the beta mixture parameters, but we find $D_j(\lambda)$ will be maximized for the j^* with the largest value of b_{j^*}. We then define this unique index (Ω) such that $D_\Omega \geq D_j$ for all values of j. This is reasonable as, decreasing the beta shape parameter a and increasing the beta shape parameter b both drive the mean of a beta distribution towards zero, which would have the greatest likelihood in a mixture of betas when the number of spillovers is large.

Using this observation, we define an upper and lower bound on the limit from Eq. S5.13 when λ is sufficiently large, such that

$$\sum_{k=1}^{K} \lim_{\lambda\to\infty} \frac{\omega_k T_k(\lambda)}{D_\Omega(\lambda)} \leq \sum_{k=1}^{K} \lim_{\lambda\to\infty} \frac{\omega_k T_k(\lambda)}{\sum_{j=1}^{K} \omega_j D_j(\lambda)} \leq \sum_{k=1}^{K} \lim_{\lambda\to\infty} \frac{\omega_k T_k(\lambda)}{\omega_\Omega D_\Omega(\lambda)} \tag{S5.19}$$

For the upper bound term, we substitute the expressions for the numerator and denominator, so our upper bound limit can be expressed as

$$\begin{gathered} \sum_{k=1}^{K} \lim_{\lambda\to\infty} \frac{\omega_k \mathcal{M}(a_k, a_k + b_k, -\lambda(cT_F + T_P))}{\sum_{j=1}^{K} \omega_j \mathcal{M}(a_j, a_j + b_j, -\lambda T_P)} \leq \\ \sum_{j=1}^{K} \lim_{\lambda\to\infty} \frac{\omega_k \mathcal{M}(a_k, a_k + b_k, -\lambda(cT_F + T_P))}{\omega_\Omega \mathcal{M}(a_\Omega, a_\Omega + b_\Omega, -\lambda T_P)} \end{gathered} \tag{S5.20}$$

Using the asymptotic form of the confluent hypergeometric function and the fact that $a_\Omega \leq a_k$ for all values of k, we see that every limit term within the sum will be zero, except when $k \in j^*$, and applying these results, we can rewrite and compute the limit such that

$$\begin{gathered} \sum_{k=1}^{K} \lim_{\lambda\to\infty} \frac{\omega_k \mathcal{M}(a_k, a_k + b_k, -\lambda(cT_F + T_P))}{\sum_{j=1}^{K} \omega_j \mathcal{M}(a_j, a_j + b_j, -\lambda T_P)} \leq \\ \left(\frac{T_P}{cT_F + T_P}\right)^{a_\Omega} \sum_{k\in j^*} \frac{\omega_k \Gamma(b_\Omega)\Gamma(a_k + b_k)}{\omega_\Omega \Gamma(b_k)\Gamma(a_\Omega + b_\Omega)} = \\ \frac{1}{(c\frac{T_F}{T_P} + 1)^{a_\Omega}} \sum_{k\in j^*} \frac{\omega_k \Gamma(b_\Omega)\Gamma(a_k + b_k)}{\omega_\Omega \Gamma(b_k)\Gamma(a_\Omega + b_\Omega)} \end{gathered} \tag{S5.21}$$

The lower bound can be computed similarly, such that

$$\frac{1}{(c\frac{T_F}{T_P}+1)^{a_\Omega}}\sum_{k\in j^*}\frac{\omega_k\Gamma(a_k+b_k)\Gamma(b_\Omega)}{\Gamma(a_\Omega+b_\Omega)\Gamma(b_k)}\leq\sum_{k=1}^{K}\lim_{\lambda\to\infty}\frac{\omega_k\mathcal{M}(a_k,a_k+b_k,-\lambda(cT_F+T_P))}{\sum_{j=1}^{K}\omega_j\mathcal{M}(a_j,a_j+b_j,-\lambda T_P)} \tag{S5.22}$$

Thus, when there is a single smallest value of a_j, the lower and upper bounds are exactly equal. For this case, we have an exact analytical solution for the limit when the prior distribution follows a mixture of beta distributions, such that

$$P_\infty(H_F>0|\bullet,H_P=0)=1-\frac{1}{(c\frac{T_F}{T_P}+1)^{a_\Omega}} \tag{S5.23}$$

When there is not a unique smallest value of a_j, the lower and upper bounds are not necessarily equal, so we instead compute analytical bounds for the limit. Since we are interested in the probability of at least one host jump in the future, we only consider the upper bound on this probability as a conservative approach. To do this, we substitute our analytically computed lower bound, which gives

$$P_\infty(H_F>0|\bullet,H_P=0)=1-\sum_{k=1}^{K}\lim_{\lambda\to\infty}\frac{\omega_k\mathcal{M}(a_k,a_k+b_k,-\lambda(cT_F+T_P))}{\sum_{j=1}^{K}\omega_j\mathcal{M}(a_j,a_j+b_j,-\lambda T_P)} \tag{S5.24}$$

$$\leq 1-\frac{1}{(cT+1)^{a_\Omega}}\sum_{k\in j^*}\frac{\omega_k\Gamma(a_k+b_k)\Gamma(b_\Omega)}{\Gamma(a_\Omega+b_\Omega)\Gamma(b_k)} \tag{S5.25}$$

The gamma functions in this expression are strictly positive because parameters ω_k, a_k, b_k, and c are all greater than zero for all values of k, every term, we notice

$$\frac{1}{(cT+1)^{a_\Omega}}\sum_{k\in j^*}\frac{\omega_k\Gamma(a_k+b_k)\Gamma(b_\Omega)}{\Gamma(a_\Omega+b_\Omega)\Gamma(b_k)}>0 \tag{S5.26}$$

Therefore, we have demonstrated that, when there is a linear correlation between the number of spillover events in the past and future, the probability of a host jump saturates to a value between zero and one as the rate of spillover approaches infinite values, and this is valid for any mixture of beta distributions. We suspect that this value is still equivalent to Eq. S5.23, but we have not shown this analytically.

References

1. Abramowitz M, Stegun IA. Handbook of mathematical functions with formulas, graphs, and mathematical tables. vol. 55. US Government printing office; 1968.
