# Supplementary material for: Pathogen host jump risk is not predicted by spillover rate, but rather by novelty
Source: PLoS Biol. 2026 Mar 19;24(3):e3003640. doi: 10.1371/journal.pbio.3003640 (PMC13001934; doi:10.1371/journal.pbio.3003640)
Supplement: S6 Text — (PDF) [file pbio.3003640.s006.pdf]

S6 Text. Evaluating reasonable prior hyper-parameters

In the main text we chose parameters for our prior distributions to broadly characterize three representative scenarios. Here, we more carefully focus on defining the shape of our prior on ϕ by establishing reasonable values for the parameters, a and b.

One possible approach to constructing a reasonable prior on ϕ would be to choose values for the beta shape parameters a and b such that the mean of the prior is equal to some estimate ($\bar{\phi}$). Host jumps are rare relative to the frequency of spillover events [1], implying that the mean of our prior should be small. A non-mixture beta distribution can take four general shapes, which arise under the following combinations of parameters a and b:

1. $a \geq 1,\ b \geq 1$
2. $a \geq 1, b < 1$
3. $a < 1,\ b < 1$
4. $a < 1, b \geq 1$

In case 1, the prior is "hump-shaped", with a peak at some intermediate value between zero and one. Because we believe the mean of this distribution should be close to zero, the parameter b must be much larger than a. Consequently, the variance will also be small, resulting in a prior that approaches a Dirac-delta function centered on $\bar{\phi}$ for large values of b. Such a prior would suggest that all pathogens are equally likely to host jump given a single spillover event, which we don't believe to be applicable in most cases.

Case 2 produces a left-skewed distribution, wherein most pathogens have host jump probability close to one. Rationally this can not be the case, as most spillover events do not result in host jumps. Therefore, between cases 1 and 2, we expect the parameter a will often be less than one because of the expectation that non-native pathogens are unlikely to transmit effectively in a novel host.

Case 3 generates a prior distribution that is "U-shaped", and the parameter a must be much smaller than b in order to have a sufficiently small mean. However, this prior suggests that there are some pathogens that will almost certainly host jump given a spillover event. Indeed, some pathogens are much more likely to host jump than others, but stochastic effects and other demographic complexities make such high host jump probabilities unlikely, even for pathogens with a large R_0.

In case 4, the prior is right-skewed, such that most pathogens are unlikely to complete a host jump. Because host jumps are thought to be rare, stochastic events, this would seem like the most reasonable shape for a prior using a single beta distribution. In this case, the suspected small mean value of ϕ can be achieved by decreasing the value of a or increasing the value of b. However, there are multiple combinations of a and b that produce equal means, so we further evaluate how changing these parameter values while retaining a constant mean would affect our conclusions.

Lastly, we note that the observations for these four cases do not necessarily apply to their usage in a mixture of beta mixture distributions, as different combinations may be able to better capture variation between pathogens than a single beta distribution alone. However, as we have shown in Supporting information S5 Text, the limiting behavior of our model when using a beta mixture prior depends only on the beta component that has the smallest value for the parameter a. Thus even when a beta mixture contains a beta from cases 1-3, its behavior as spillover rate goes to infinity will be determined by the behavior of case 4.

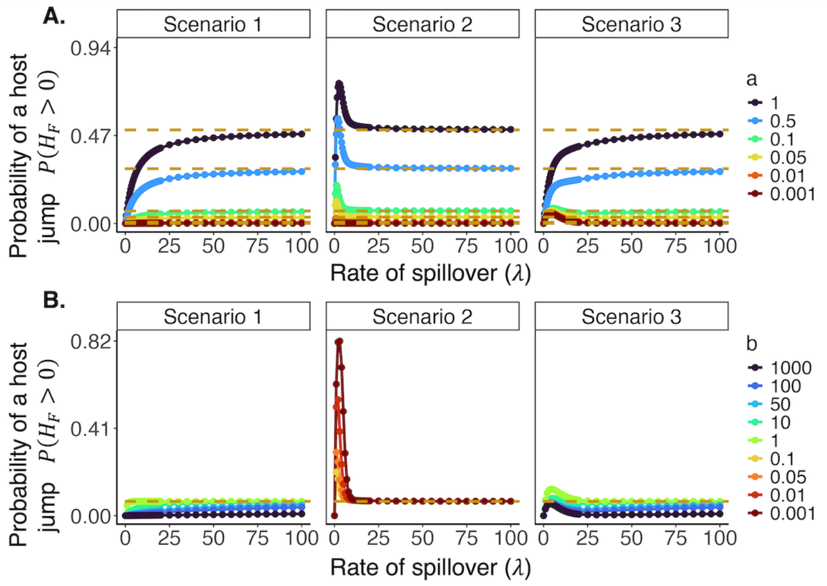

Fig S6.1. Model trajectories over varying prior hyperparameters. Here we show the effects of changing the values of different prior hyper-parameters in the three scenarios discussed in the main text. Results for different values of the parameter a are shown in panel **A** and results for different values of the parameter b are shown in panel **B**. For the beta mixture distribution (i.e., scenario 3, only the shape parameters in the first term of the mixture (i.e., $0.8 \cdot \text{Beta}(a = 0.1,\ b = 10)$) are changed. In panel **A**, we see that changing the value of a affects the limit value in each of these scenarios, but all curves converge at approximately similar rates. In contrast, we see that changing the value of b in panel **B** does not change the limit value, but the curves take longer to saturate for large values of b. Note that not all values of b are used in each scenario in panel **B**. Scenarios 1 and 3 do not use $b < 1$ to avoid redundancy with scenario 2. Similarly, scenario 2 does not use $b \geq 1$, as this would yield a left-skewed prior, which is not biologically realistic. For each panel, the remaining parameter values are $T_P = 1$, $T_F = 1$, $c = 1$. The code needed to generate this figure can be found in https://doi.org/10.5281/zenodo.14154724

Results under changing prior hyper-parameters

Since a prior distribution with a reasonable mean can be achieved by changing both a and b, we would like to characterize the effects of changing the values of these parameters. As we demonstrated in Supporting information S5 text, as the spillover rate goes to infinity, the probability of a host jump converges to a value between zero and one. The precise value that it converges on only depends on the parameter a, the slope c of the linear relationship between the rate of past and future spillover events, and the size of the past and future spillover windows (T_P and T_F). Therefore, as the rate of spillover gets large, decreasing the value of a will result in a decrease in the probability of a host jump, but decreases in the value of b will have no impact on that probability. However, as we show below, increasing the value of b decreases the rate at which convergence is achieved. We show the difference between changing either a or b in Fig. S6.1.

We also quantify the rate of convergence using a standardized distance metric under different values of a and b, shown in Fig. S6.2.

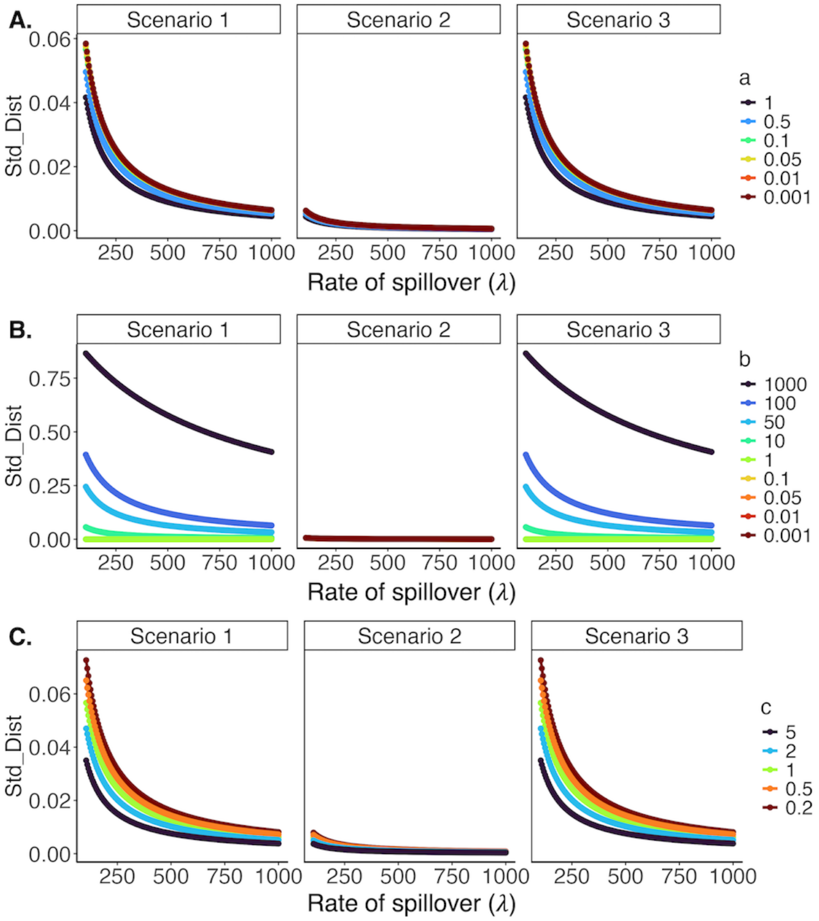

Fig S6.2. Standardized distance from convergence limit as a function of several model parameters. Here we show how changing the values of our model parameters in the three scenarios from the main text affect the relative distance from convergence. Results are shown for different values of a (panel **A**), b (panel **B**), and c (panel **C**). Note that the y-axis values differ substantially between panels. For the beta mixture distribution in scenario 3, only the shape parameters in the first term of the mixture (i.e., $0.8 \cdot \mathrm{Beta}(a = 0.1,\ b = 10)$) are changed. We see that the distance from convergence is relatively insensitive to changes in the value of parameters a and c. However, this distance is highly sensitive to increasing values of b, as our standardized distance metric is greatest when b is large. Importantly, we note that this same trend is unlikely to hold when increasing a or c, as we see here that increasing these values leads to faster convergence. For each panel, the remaining parameter values are $T_P = 1$, $T_F = 1$, and $c = 1$ when not otherwise specified. The code needed to generate this figure can be found in https://doi.org/10.5281/zenodo.14154724

We define this distance metric metric as:

$$Std\_Dist = \frac{|\mathcal{F}(N) - \mathcal{L}|}{\mathcal{L}} \tag{S6.1}$$

where $\mathcal{F}(N)$ is the analytical solution to our model along the $c:1$ line, and $\mathcal{L}$ is the value of the limit derived in Supporting information S5 Text. In general, we see that this metric is relatively insensitive to changes to the parameters a and c for any given scenario; however, the metric is highly sensitive to the value of b. In scenarios 1 and 3 in

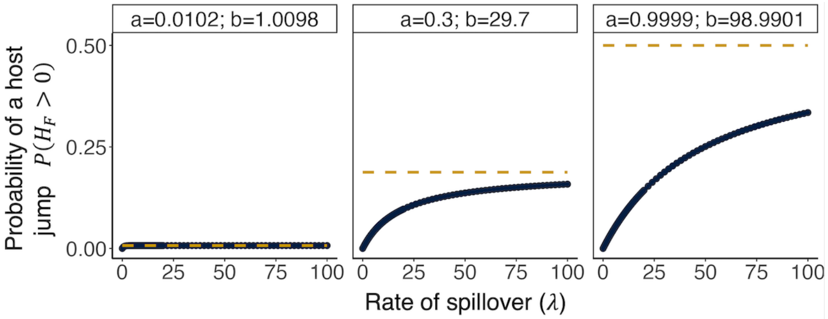

Fig S6.3. Effects of changing hyper-parameters of right-skewed prior. Here we show how changing the values of the prior hyper-parameters for a right-skewed prior (like scenario 1) would affect our results. In all cases, we see that these curves all eventually converge to a value between zero and on depending on the value of a, and that the speed at which they converge to this value depends on b. Parameters are chosen such that all three priors used here have equal means of $\bar{\phi} = 0.01$. When a is small, b must be close to one, so the probability of a future host jump converges to a small value quickly. As the values of a and b increase, the probability of a future host jump increases while the rate of convergence decreases. Note that the risk of a host jump increases monotonically with spillover rate for right skewed priors, as we have already shown in the main text. For each panel, the remaining parameter values are $T_P = 1$, $T_F = 1$, $c = 1$. The code needed to generate this figure can be found in https://doi.org/10.5281/zenodo.14154724

Fig. S6.2**B**, we see that increasing values of b leads to larger values for our distance metric, implying that convergence is slower for large values of b, so a pathogen's absolute rate of spillover must be increasingly large to be considered 'high spillover' and convergent. In other words, increasing the value of b suggests that less information is gained from each spillover event, than when b is smaller. From this figure, we also see that the distance from our convergence metric is almost completely insensitive to changes in the parameters a and c.

We also explore the effects of changing the values of a and b while retaining a fixed mean for the prior distribution. We focus specifically on the right-skewed prior (similar to scenario 1 in the main text). We define a conservative hypothetical mean for our prior such that $\bar{\phi} = 0.01$, and restrict the prior parameters such that $a < 1$ and $b \geq 1$. We use three sets of parameters to broadly characterize possible results within this parameter space and define our three right skewed priors as:

$$\pi(\phi) \sim \text{Beta}(a = 0.0102,\ b = 1.0098) \tag{S6.2}$$

$$\pi(\phi) \sim \text{Beta}(a = 0.3,\ b = 29.7) \tag{S6.3}$$

$$\pi(\phi) \sim \text{Beta}(a = 0.9999,\ b = 98.9901) \tag{S6.4}$$

We show our model predictions under these different prior parameterizations in Fig. S6.3. As expected from the results in Fig. S6.1, the limiting values of host jump risk depends on the value of a, and the rate at which this level of risk is achieved depends on b. While the conclusion as to which types of pathogens are most likely to host jump is the same for each of these priors, the magnitude of the risk is fundamentally different. While all three priors have the same mean, this dramatic difference in the magnitude of risk posed by zoonotic pathogens highlights the importance of estimating values for our prior parameters for pandemic prediction.

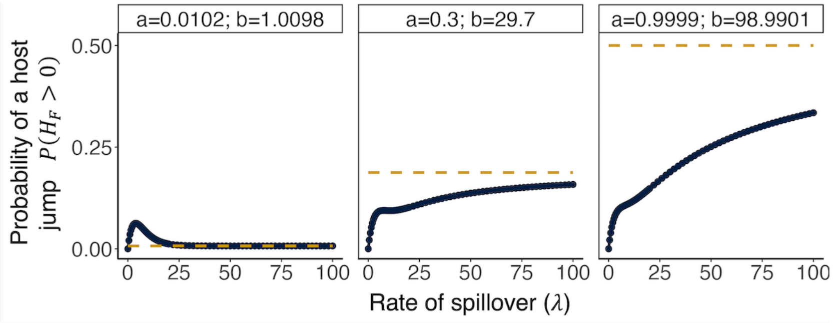

Fig S6.4. Effects of changing hyper-parameters of right-skewed mixture component. Here we show how changing the values of the prior hyper-parameters for the right-skewed component of our beta mixture prior (like scenario 3) would affect our results. We see that the parameters of the right skewed component control the value and rate of convergence. However, unlike our results for the mixture distribution in the main text (Scenario 3), it is not always the case that pathogens with intermediate levels of spillover pose the greatest host jump risk. Instead, when the parameter a is sufficiently large, pathogens that spill over most pose the greatest host jump risk. For each panel, the remaining parameter values are $T_P = 1$, $T_F = 1$, $c = 1$. The code needed to generate this figure can be found in https://doi.org/10.5281/zenodo.14154724

In addition to the single beta distributions, we also use these parameters in a mixture distribution, similar to Scenario 3 in the main text, where our prior has the form $\pi(\phi) \sim 0.8 \cdot \text{Beta}(a,\ b) + 0.2 \cdot \text{Beta}(100,\ 400)$, where the first component in the mixture is replaced with each of the three aforementioned right-skewed beta distributions in Fig. S6.4. Using these different parameterizations, we see that when the parameter a is small, we see that host jump risk is highest for intermediate levels of spillover due to the subset of "higher-risk pathogens" represented by the second component of the mixture. However, when the value of a is sufficiently large in the right-skewed component, the subset of "high risk pathogens" characterized by the second mixture component are "out-competed" by pathogens that frequently spill over in terms of host jump risk. Therefore, pathogens that frequently spill over are most likely to host jump in these cases.

Based on these results for different prior parameterizations, we see that our choices for the values of a and b play a significant role in quantifying the risk posed by zoonotic pathogens. The shape of our prior distribution (and the parameters that define it) will depend heavily on our understanding of characteristics of pathogens, the hosts, and the environment that either facilitate or hinder the process of a successful host jump. Past and future research on these subjects will be critical to characterizing a prior on ϕ that allows us to accurately evaluate the relationship between spillover and host jump risk and identify contexts where host jumps are most likely.

References

1. Parrish CR, Holmes EC, Morens DM, Park EC, Burke DS, Calisher CH, et al. Cross-species virus transmission and the emergence of new epidemic diseases. Microbiology and Molecular Biology Reviews. 2008;72(3):457-70.
